# A large 28S rDNA-based phylogeny confirms the limitations of established morphological characters for classification of proteocephalidean tapeworms (Platyhelminthes, Cestoda)

**DOI:** 10.3897/zookeys.500.9360

**Published:** 2015-04-27

**Authors:** Alain de Chambrier, Andrea Waeschenbach, Makda Fisseha, Tomáš Scholz, Jean Mariaux

**Affiliations:** 1 Natural History Museum of Geneva, CP 6434, CH - 1211 Geneva 6, Switzerland; 2 Natural History Museum, Life Sciences, Cromwell Road, London SW7 5BD, UK; 3 Institute of Parasitology, Biology Centre of the Czech Academy of Sciences, Branišovská 31, 370 05 České Budějovice, Czech Republic; 4 Department of Genetics and Evolution, University of Geneva, CH - 1205 Geneva, Switzerland

**Keywords:** Eucestoda, Proteocephalidae, systematics, molecular phylogeny, host-parasite associations, *Spasskyellina*

## Abstract

Proteocephalidean tapeworms form a diverse group of parasites currently known from 315 valid species. Most of the diversity of adult proteocephalideans can be found in freshwater fishes (predominantly catfishes), a large proportion infects reptiles, but only a few infect amphibians, and a single species has been found to parasitize possums. Although they have a cosmopolitan distribution, a large proportion of taxa are exclusively found in South America. We analyzed the largest proteocephalidean cestode molecular dataset to date comprising more than 100 species (30 new), including representatives from 54 genera (80%) and all subfamilies, thus significantly improving upon previous works to develop a molecular phylogeny for the group. The Old World origin of proteocephalideans is confirmed, with their more recent expansion in South America. The earliest diverging lineages are composed of Acanthotaeniinae and Gangesiinae but most of the presently recognized subfamilies (and genera) appear not to be monophyletic; a deep systematic reorganization of the order is thus needed and the present subfamilial system should be abandoned. The main characters on which the classical systematics of the group has been built, such as scolex morphology or relative position of genital organs in relation to the longitudinal musculature, are of limited value, as demonstrated by the very weak support for morphologically-defined subfamilies. However, new characters, such as the pattern of uterus development, relative ovary size, and egg structure have been identified, which may be useful in defining phylogenetically well-supported subgroups. A strongly supported lineage infecting various snakes from a wide geographical distribution was found. Although several improvements over previous works regarding phylogenetic resolution and taxon coverage were achieved in this study, the major polytomy in our tree, composed largely of siluriform parasites from the Neotropics, remained unresolved and possibly reflects a rapid radiation. The genus *Spasskyellina* Freze, 1965 is resurrected for three species of *Monticellia* bearing spinitriches on the margins of their suckers.

## Introduction

Proteocephalideans (Platyhelminthes: Cestoda) form a morphologically homogeneous group of tapeworms found worldwide in freshwater fishes, reptiles, and amphibians (a single species is known from marsupial mammals). To our knowledge 315 valid species are currently known (unpublished), a large proportion of them being parasites of South American siluriform fishes (Freze 1965, Rego 1994).

Proteocephalideans historically formed their own order (Proteocephalidea with only one family, Proteocephalidae), the monophyly of which is strongly supported, but recent molecular analyses have placed them within a paraphyletic assemblage of ‘hooked’ tetraphyllidean cestodes (formerly Onchobothriidae), parasites of sharks and rays, which has led to the erection of a new order, the Onchoproteocephalidea by Caira et al. (2014). However, the lack of any morphological synapomorphies for this new order made this a somewhat controversial decision. For the purpose of the present paper, which is to study the internal relationships of the “terrestrial” onchoproteocephalideans (= proteocephalideans), this point is marginal and the new scheme proposed by Caira et al. (2014) is not considered further.

Previous attempts to study the interrelationships of proteocephalideans resulted in overall poorly resolved phylogenies. At the morphological level, the difficulty of defining reliable informative characters has prevented the construction of a stable taxonomic arrangement of the group (Rego 1994, 1995). The traditionally accepted families Proteocephalidae and Monticelliidae have been abandoned, and the whole group has been split into a number of subfamilies and genera, including the type genus *Proteocephalus* Weinland, 1858, which are sometimes obviously artificial because of their lack of synapomorphies and diversity of life-history traits (see de Chambrier et al. 2004c, 2009a). Molecular studies that have tried to resolve the proteocephalidean tree topology have largely been based on the variable domains (D1–D3) of the large nuclear ribosomal RNA subunit (28S rDNA), using increasingly larger datasets, i.e. 54 taxa analyzed by Zehnder and Mariaux (1999), and 75 taxa by de Chambrier et al. (2004c). Hypša et al. (2005) analyzed the phylogenetic relationships of only 52 taxa, but used sequences of three ribosomal RNA genes and the internal transcribed spacer 2 (ITS2). Additional molecular studies mostly considered questions at the specific/generic level [e.g. the *Proteocephalus* aggregate (Scholz et al. 2007); African *Proteocephalus* (de Chambrier et al. 2011); *Testudotaenia* Freze, 1965 (de Chambrier et al. 2009a), Corallobothriinae (Rosas-Valdez et al. 2004, Scholz et al. 2011)] or employed only a very limited taxon sampling (e.g. Zehnder and de Chambrier 2000, Škeříková et al. 2001, de Chambrier et al. 2008, Scholz et al. 2013).

Although these studies have allowed for a better understanding of relationships within and between several subgroups, the major nodes of the proteocephalidean tree remain poorly supported, especially when considering the South American lineages. In the present contribution, an unprecedented collection of proteocephalidean samples have been gathered that includes the majority of all valid genera (54 out of 67), thus significantly increasing the taxon sampling within the order and adding representatives from previously unrepresented subfamilies. 28S rDNA sequences homologous to those published in studies by Zehnder and Mariaux (1999) and de Chambrier et al. (2004c) have been generated, and the newly generated data has been analyzed in conjunction with those previously published. Thus, the 28S rDNA data presented here represent the most comprehensive sampling of proteocephalideans to date.

## Methods

### Taxon sampling

The present study is based on the evaluation of a dataset of proteocephalideans collected during long-term studies carried out by the present authors and their co-workers, especially as part of research activities linked to the NSF-PBI project “A Survey of the Tapeworms (Cestoda: Platyhelminthes) from Vertebrate Bowels of the Earth” (2008–2014), which was aimed at mapping the global diversity of tapeworms. Despite significant sampling effort covering all zoogeographical regions and the most important host groups, the number of studied proteocephalideans that parasitize amphibians remains relatively small due to the paucity of cestodes in these hosts. In addition, several newly described proteocephalideans from the southern part of the Neotropical Region (Argentina) were not available for molecular studies. Among the 13 proteocephalidean genera that are not represented in our sampling, none presently contains more than two species (see Caira et al. 2012).

All taxa considered in this study are listed in Table 1. Most taxa included in de Chambrier et al. (2004c) are included in the present analysis; however, some taxonomical changes and novel identifications have taken place since this paper was published: *Proteocephalus pirarara* (Woodland, 1935a) is now *Scholzia emarginata* (Diesing, 1850); Ophiotaenia cf. gallardi is now *Ophiotaenia* sp.; *Pseudocrepidobothrium* sp. is now *Pseudocrepidobothrium ludovici* Ruedi & de Chambrier, 2012; *Megathylacus brooksi* Rego & Pavanelli, 1985 is now *Megathylacus jandia* (Woodland, 1934b); Spatulifer cf. maringaensis is now *Spatulifer maringaensis* Pavanelli & Rego, 1989. All but five molecular samples are vouchered, and in 86% of cases the vouchers are the hologenophore (sensu Astrin et al. 2013).

**Table 1.** Taxa used in the current study. Voucher numbers refer to the collections of the Natural History Museum of Geneva (MHNG-PLAT); Larry R Penner Parasitology Collection, Storrs, Connecticut, USA (LRP); Collección Nacional de Helminthos, México (CNHE); Collections of the Institute of Parasitology of the Czech Academy of Sciences (IPCAS). Out.: Outgroup. Type species are marked with a (T) and hologenophores with an *.

Species	Host species	Voucher number	Accession Number	Reference	Surface ovary %
*Acanthotaenia shipleyi* (T)	*Varanus salvator*	*MHNG-PLAT-32887	AJ583453	de Chambrier et al. 2004c	6.8
*Ageneiella brevifilis* (T)	*Ageneiosus inermis*	*MHNG-PLAT-21841	AJ388600	Zehnder et al. 1999	11.2
*Amphoteromorphus ninoi*	*Brachyplatystoma filamentosum*	*MHNG-PLAT-22239	AJ388624	de Chambrier et al. 2004c	11.7
*Amphoteromorphus peniculus* (T)	*Brachyplatystoma rousseauxii*	*MHNG-PLAT-60052	KP729410	This paper	12.3
*Amphoteromorphus piraeeba*	*Brachyplatystoma filamentosum*	MHNG-PLAT-22227	KP729407	This paper	12.5
*Amphoteromorphus piriformis*	*Brachyplatystoma roussseauxii*	*MHNG-PLAT-22211	AJ275231	de Chambrier et al. 2004c	12.5
*Australotaenia bunthangi*	*Enhydris enhydris*	*MHNG-PLAT-75447	KP729409	This paper	5.0
*Barsonella lafoni* (T)	*Clarias gariepinus*	*MHNG-PLAT-49399	FM955143	de Chambrier et al. 2009b	11.5
*Brayela karuatayi* (T)	*Platynematichthys notatus*	*MHNG-PLAT-63128	KP729406	This paper	10.9
*Brooksiella praeputialis* (T)	*Cetopsis coecutiens*	*MHNG-PLAT-21996	AJ275229	de Chambrier et al. 2004c	17.3
*Cangatiella arandasi* (T)	*Trachelyopterus galeatus*	*MHNG-PLAT-34736	KP729411	This paper	8.0
*Choanoscolex abcisus* (T)	*Pseudoplatystoma corruscans*	*MHNG-PLAT-17905	AJ388630	Zehnder et al. 1999	12.8
*Choanoscolex* sp.	*Pseudoplatystoma fasciatum*	*MHNG-PLAT-25102	AJ275064	de Chambrier et al. 2004c	5.1
*Corallobothrium solidum* (T)	*Malapterurus electricus*	*MHNG -PLAT-31553	AJ583450	de Chambrier et al. 2004c	7.2-7.4
Corallobothrium cf. solidum	*Malapterurus gossei*	*MHNG-PLAT-63117	JN005780	Scholz et al. 2011	11.0
*Corallotaenia intermedia*	*Ictalurus punctatus*	*MHNG-PLAT-25795	AJ275232	de Chambrier et al. 2004c	11.3
*Crepidobothrium gerrardii* (T)	*Boa constrictor*	*MHNG-PLAT-66546	KC786018	Scholz et al. 2013	3.6
*Electrotaenia malopteruri* (T)	*Malapterurus electricus*	*MHNG-PLAT-33995	JX477434	Ash et al. 2012	4.6-5.2
*Endorchis piraeeba* (T)	*Brachyplatystoma filamentosum*	*MHNG-PLAT-21738	AJ388603	Zehnder et al. 1999	5.9
*Ephedrocephalus microcephalus* (T)	*Phractocephalus hemioliopterus*	*MHNG-PLAT-21910	AJ388605	Zehnder et al. 1999	11.4
*Essexiella fimbriata* (T)	*Ictalurus balsanus*	CNHE 4217	AY548162	Rosas Valdez et al. 2004	15.1
*Gangesia agraensis*	*Wallago attu*	*MHNG-PLAT-75457	JX477443	Ash et al. 2012	16.4
*Gangesia parasiluri*	*Silurus asotus*	*MHNG-PLAT-22436	AF286935	Olson et al. 2001	15.0
*Gibsoniela mandube* (T)	*Ageneiosus* sp.	*MHNG-PLAT-63119	KP729412	This paper	8.6
*Gibsoniela meursaulti*	*Ageneiosus inermis*	*MHNG-PLAT-21839	AJ388631	Zehnder et al. 1999	12.3
*Glanitaenia osculata* (T)	*Silurus glanis*	N/A	AJ388619	Zehnder et al. 1999	11.1
*Goezeella siluri* (T)	*Pinirampus pirinampu*	*MHNG-PLAT-21877	AJ388612	Zehnder et al. 1999	11.9
*Harriscolex kaparari* (T)	*Pseudoplatystoma tigrinum*	*MHNG-PLAT-22018	AJ275227	de Chambrier et al. 2004c	13.7
*Houssayela sudobim* (T)	*Sorubimichthys planiceps*	*MHNG-PLAT-62586	KP729404	This paper	9.7
*Jauella glandicephalus* (T)	*Zungaro jahu*	*MHNG-PLAT-31179	KP729399	This paper	9.6
*Kapsulotaenia* sp. 1	*Varanus rosenbergi*	*MHNG-PLAT-32842	AJ583452	de Chambrier et al. 2004c	5.5
*Kapsulotaenia* sp. 2	*Varanus gouldii*	*MHNG-PLAT-32839	AJ583455	de Chambrier et al. 2004c	3.5
*Kapsulotaenia* sp. 4	*Varanus varius*	*MHNG-PLAT-32838	AJ583454	de Chambrier et al. 2004c	6.5
*Macrobothriotaenia ficta* (T)	*Xenopeltis unicolor*	*MHNG-PLAT-75454	KC786020	Scholz et al. 2013	4.1
*Manaosia bracodemoca* (T)	*Sorubim lima*	*MHNG-PLAT-34186	KP729414	This paper	16.4
*Marsypocephalus heterobranchus*	*Heterobranchus bidorsalis*	*MHNG-PLAT-62973	KP729408	This paper	7.3
*Marsypocephalus rectangulus* (T)	*Clarias anguillaris*	*MHNG-PLAT-49366	KP729405	This paper	11.0
*Megathylacoides giganteum* (T)	*Ictalurus dugesi*	N/A	AY307117	Rosas Valdez et al. 2004	15.1
*Megathylacoides lamothei*	*Ictalurus furcatus*	CNHE 4889	AY548165	Rosas Valdez et al. 2004	13.8
*Megathylacoides* sp.	*Ictalurus punctatus*	*MHNG-PLAT-35373	FM956086	de Chambrier et al. 2009a	9.4
*Megathylacus jandia* (T)	*Zungaro zungaro*	*MHNG-PLAT-21874	AJ388596	Zehnder et al. 1999	8.6
*Monticellia coryphicephala* (T)	*Salminus brasiliensis*	*MHNG-PLAT-17984	AJ238832	Zehnder et al. 1999	18.5
*Nomimoscolex admonticellia*	*Pinirampus pirinampu*	*MHNG-PLAT-21870	AJ388628	Zehnder et al. 1999	7.1
*Nomimoscolex chubbi*	*Gymnotus carapo*	*MHNG-PLAT-20351	AJ388625	Zehnder et al. 1999	7.7-12.4
*Nomimoscolex dorad*	*Brachyplatystoma rousseauxii*	*MHNG-PLAT-22269	AJ388613	Zehnder et al. 1999	7.5
*Nomimoscolex lenha*	*Sorubimichthys planiceps*	*MHNG-PLAT-21740	AJ388611	Zehnder et al. 1999	9.8
*Nomimoscolex lopesi*	*Pseudoplatystoma fasciatum*	*MHNG-PLAT-21963	AJ388618	Zehnder et al. 1999	8.8
*Nomimoscolex matogrossensis*	*Hoplias malabaricus*	*MHNG-PLAT-17913	AJ388614	Zehnder et al. 1999	12.2-14.5
*Nomimoscolex piraeeba* (T)	*Brachyplatystoma capapretum*	*MHNG-PLAT-22284	AJ388608	Zehnder et al. 1999	10.6-12.8
*Nomimoscolex sudobim*	*Pseudoplatystoma fasciatum*	*MHNG-PLAT-21969	AJ388597	Zehnder et al. 1999	12.0
*Nomimoscolex suspectus*	*Brachyplatystoma vaillanti*	*MHNG-PLAT-22298	AJ388602	de Chambrier et al. 2004c	6.2-10.2
*Nupelia portoriquensis* (T)	*Sorubim lima*	*MHNG-PLAT-34185	KP729401	This paper	10.3
*Ophiotaenia bungari*	*Bungarus fasciatus*	*MHNG-PLAT-75442	KC786022	Scholz et al. 2013	3.1
*Ophiotaenia europaea*	*Natrix maura*	*MHNG-PLAT-18407	AJ388598	Zehnder et al. 1999	12.7
*Ophiotaenia filaroides*	*Ambystoma tigrinum*	*MHNG-PLAT-63372	KP729416	This paper	11.5
*Ophiotaenia gallardi*	*Pseudechis porphyriacus*	*MHNG-PLAT-36550	KC786025	Scholz et al. 2013	3.2
*Ophiotaenia grandis*	*Agkistrodon piscivorus*	N/A	AJ388632	Zehnder et al. 1999	2.1
*Ophiotaenia jarara*	*Bothrops jararaca*	*MHNG-PLAT-12393	AJ388607	Zehnder et al. 1999	2.4
*Ophiotaenia lapata*	*Madagascarophis colubrina*	*MHNG-PLAT-79567	KC786021	Scholz et al. 2013	2.8
*Ophiotaenia ophiodex*	*Causus maculatus*	*MHNG-PLAT-25962	AJ388620	Zehnder et al. 1999	4.2
*Ophiotaenia paraguayensis*	*Hydrodynastes gigas*	*MHNG-PLAT-16927	AJ388629	Zehnder et al. 1999	3.3
Ophiotaenia cf. perspicua	*Nerodia rhombifer*	*MHNG-PLAT-35370	KP729415	This paper	2.3
*Ophiotaenia sanbernardinensis*	*Helicops leopardinus*	*MHNG-PLAT-18251	AJ388637	Zehnder et al. 1999	5.0
*Ophiotaenia saphena*	*Lithobates pipiens*	*MHNG-PLAT-32851	KP729402	This paper	8.3-8.7
*Pangasiocestus romani* (T)	*Pangasius larnaudii*	*MHNG-PLAT-75449	KP729397	This paper	10.6
*Paraproteocephalus parasiluri* (T)	*Silurus asotus*	*MHNG-PLAT-22438	AJ388604	Zehnder et al. 1999	4.3
*Peltidocotyle lenha*	*Zungaro zungaro*	*MHNG-PLAT-22373	AJ238837	Zehnder et al. 1999	14.7
*Peltidocotyle rugosa* (T)	*Pseudoplatystoma reticulatum*	*MHNG-PLAT-22374	AJ238835	Zehnder et al. 1999	13.9-14.7
*Postgangesia inarmata*	*Silurus glanis*	*MHNG-PLAT-34212	AM931032	de Chambrier et al. 2008	12.5
Proteocephalidae gen. sp.	*Amia calva*	*MHNG-PLAT-35548	FM956088	de Chambrier et al. 2009a	9.3
*Proteocephalus filicollis*	*Gasterosteus aculeatus*	*MHNG-PLAT-24081	AJ388636	Zehnder et al. 1999	16.3
*Proteocephalus fluviatilis*	*Micropterus dolomieu*	IPCAS C-364	KP729390	This paper	17.0
*Proteocephalus glanduligerus*	*Clarias* sp.	*MHNG-PLAT-50013	KP729392	This paper	9.8
*Proteocephalus gobiorum*	*Neogobius fluviatilis*	IPCAS C-299	KP729393	This paper	19.7
*Proteocephalus hemioliopteri*	*Phractocephalus hemioliopterus*	*MHNG-PLAT-21889	AJ388622	Zehnder et al. 1999	11.8
*Proteocephalus kuyukuyu*	*Pterodoras granulosus*	*MHNG-PLAT-66572	KP729388	This paper	Immature
*Proteocephalus longicollis*	*Coregonus lavaretus*	*MHNG-PLAT-21681	AJ388626	de Chambrier et al. 2004c	13.3
*Proteocephalus macrocephalus*	*Anguilla anguilla*	N/A	AJ388609	Zehnder et al. 1999	18.3
*Proteocephalus macrophallus*	*Cichla monoculus*	MHNG-PLAT-36526	KP729394	This paper	6.0-6.6
*Proteocephalus midoriensis*	*Lefua echigonia*	MHNG-PLAT-22431	AJ388610	Zehnder et al. 1999	19.4
*Proteocephalus percae*	*Perca fluviatilis*	*MHNG-PLAT-36744	AJ388594	Zehnder et al. 1999	13.8
*Proteocephalus perplexus*	*Amia calva*	*MHNG-PLAT-35366	FM956089	de Chambrier et al. 2009a	12.0
*Proteocephalus pinguis*	*Esox lucius*	*IPCAS C-679	KP729395	This paper	9.6
*Proteocephalus plecoglossi*	*Plecoglossus altivelis*	MHNG-PLAT-22434	AJ388606	de Chambrier et al. 2004c	7.4
*Proteocephalus renaudi*	*Platydoras costatus*	*MHNG-PLAT-17894	AJ388638	Zehnder et al. 1999	7.1
*Proteocephalus sagittus*	*Barbatula barbatula*	IPCAS C-33	KP729391	This paper	13.4
*Proteocephalus sulcatus*	*Clarotes laticeps*	MHNG-PLAT-54150	KP729396	This paper	10.6
*Proteocephalus synodontis*	*Synodontis caudivittatus*	*MHNG-PLAT-62931	JN005778	Scholz et al. 2011b	9.2-13.0
*Proteocephalus tetrastomus*	*Hypomesus nipponensis*	MHNG-PLAT-22429	AJ388635	Zehnder et al. 1999	7.0-11.4
*Proteocephalus* sp.	*Ictalurus punctatus*	*MHNG-PLAT-36278	FM956085	de Chambrier et al. 2009a	11.0
*Pseudocrepidobothrium eirasi* (T)	*Phractocephalus hemioliopterus*	MHNG-PLAT-27431	AJ388623	de Chambrier et al. 2004c	11.6
*Pseudocrepidobothrium ludovici*	*Phractocephalus hemioliopterus*	*MHNG-PLAT-22108	AJ275063	Zehnder et al. 1999	9.7-10.3
*Regoella brevis* (T)	*Pseudoplatystoma reticulatum*	*MHNG-PLAT-79184	KP729389	This paper	11.5
*Ritacestus ritaii* (T)	*Rita rita*	*MHNG-PLAT-63242	JX477447	Ash et al. 2012	17.7
*Rostellotaenia nilotica* (T)	*Varanus niloticus*	*MHNG-PLAT-34195	KP729398	This paper	7.0
*Rostellotaenia* sp.	*Varanus exanthematicus*	MHNG-PLAT-25026	AJ388593	de Chambrier et al. 2004c	3.9
*Rudolphiella piracatinga*	*Calophysus macropterus*	*MHNG-PLAT-19868	AJ388627	Zehnder et al. 1999	10.4
*Rudolphiella szidati*	*Luciopimelodus pati*	*MHNG-PLAT-24668	AJ388617	de Chambrier et al. 2004c	14.4
*Sandonella sandoni* (T)	*Heterotis niloticus*	*MHNG-PLAT-49356	AM931033	Unpublished	8.8
*Scholzia emarginata* (T)	*Phractocephalus hemioliopterus*	*MHNG-PLAT-22106	KC786016	Scholz et al. 2013	10.8-15.9
*Sciadocephalus megalodiscus* (T)	*Cichla monoculus*	MHNG-PLAT-37332	KP729403	This paper	N/A
*Silurotaenia siluri* (T)	*Silurus glanis*	MHNG-PLAT-25027	AJ388592	Zehnder et al. 1999	14.8
*Spasskyellina lenha* (T)	*Sorubimichthys planiceps*	*MHNG-PLAT-69600	KP729413	This paper	9.8
*Spasskyellina spinulifera*	*Pseudoplatystoma corruscans*	*MHNG-PLAT-34216	KP729417	This paper	10.1
*Spatulifer maringaensis*	*Sorubim lima*	*MHNG-PLAT-21986	AJ388634	de Chambrier et al. 2004c	17.4
*Testudotaenia testudo* (T)	*Apalone spinifera*	*MHNG-PLAT-35320	FM956082	de Chambrier et al. 2009a	6.2
*Thaumasioscolex didelphidis* (T)	*Didelphis marsupialis*	*MHNG-PLAT-28993	AJ275065	de Chambrier et al. 2004c	8.4
*Travassiella jandia* (T)	*Zungaro jahu*	MHNG-PLAT-31175	KP729400	This paper	8.6-10.7
*Vermaia pseudotropii* (T)	*Clupisoma garua*	*MHNG-PLAT-63247	JX477453	Ash et al. 2012	3.3
*Zygobothrium megacephalum* (T)	*Phractocephalus hemioliopterus*	*MHNG-PLAT-21846	AJ388621	Zehnder et al. 1999	20.8
[Out.] *Acanthobothrium* sp.	*Dasyatis longus*	LRP-2112	AF286953	Olson et al. 2001	N/A
[Out.] *Phyllobothrium lactuca*	*Mustelus asterias*	LRP_2115	AF286960	Olson et al. 2001	N/A
[Out.] Tetraphyllidea gen. sp.	*Squalus acanthias*	N/A	AJ388591	Zehnder et al. 1999	N/A

### Molecular phylogenetic analyses

Total genomic DNA extraction, PCR amplification, and sequencing were done as outlined in Scholz et al. (2013). Eighty-three published and 30 newly generated 28S rDNA sequences were combined and analysed in conjunction (see Table 1 for GenBank accession numbers and further details). *Acanthobothrium* sp. (‘Onchoproteocephalidea’), *Phyllobothrium lactuca* Beneden, 1850 (Phyllobothriidea) and “Tetraphyllidea” gen. sp. were used as outgroup taxa. Sequences were aligned with MAFFT (Multiple Alignment using Fast Fourier Transform, http://www.ebi.ac.uk/Tools/msa/mafft/) using the default settings. An alignment mask excluding sites of uncertain positional homology was generated using ZORRO (Wu et al. 2012). ZORRO uses a pair Hidden Markov Model and a weighted sum of pairs scheme (guided by a reference tree) that sums up the probability that a given alignment column appears over the total alignment landscape, thus providing an objective estimate of whether positions consist of correctly aligned, homologous residues. Default settings were used except for the invocation of the – sample option; positions with confidence scores < 0.4 were excluded from subsequent analyses. MRMODELTEST v. 2.3 (Nylander 2004) was used to select models of sequence evolution using the Akaike Information Criterion. Bayesian inference (BI) analysis was performed using MRBAYES version 3.2 (Ronquist and Huelsenbeck 2003) using the GTR model of sequence evolution with proportion of invariant sites and gamma-distributed rate variation amongst sites (nst = 6, rates = invgamma). Default prior settings and heating schemes were used. Two parallel runs were performed for 10,000,000 generations and sampled every 1,000 generations. The burn-in was defined as the point at which the average standard deviation of split frequencies were < 0.01. Consensus trees were constructed using the 50% majority rule and nodes with < 0.95 posterior probabilities (pp) were collapsed. Leaf-stability tests, implemented in P4 (Foster 2004), were carried out to identify unstable taxa. Given a set of trees, for each set of four taxa, the frequency of the four possible resolutions of quartets was calculated. For each taxon, the highest percentages for quartets including that taxon were averaged and listed as “Maximum”. Therefore, unstable taxa across the trees were considered to be those that have lower average maximum percentages. In this study, the three taxa with the lowest “Maximum” values were eliminated from analyses in order to increase nodal support for the remaining groupings (Wilkinson 1996).

### Morphological analysis

Taxonomic identification was performed on specimens fixed and mounted on microscope slides according to de Chambrier (2001). Uterine development was characterized according to de Chambrier et al. (2004c) but a new “intermediate type” was recognized and is described below (see Fig. 2). The relative size of the ovary, i.e. the ovary to proglottid surface ratio, was calculated for each species according to the method described in de Chambrier et al. (2012). Approximate values might be due to inaccurate drawings or fixation methods reported by the original authors. Eggs were examined in distilled water.

## Data Resources

The data underpinning the analysis reported in this paper are deposited in the Dryad Data Repository at http://dx.doi.org/10.5061/dryad.dv44b.

## Results

### Molecular phylogeny

The complete 28S rDNA dataset comprised 110 ingroup taxa (from 54 genera, representing all 13 currently recognized subfamilies) and three outgroup taxa. Importantly, 46 genera were represented by their type species (see Table 1). The alignment consisted of 1937 characters of which 420 were excluded, leaving 1517 for the analyses.

**Figure 1.**
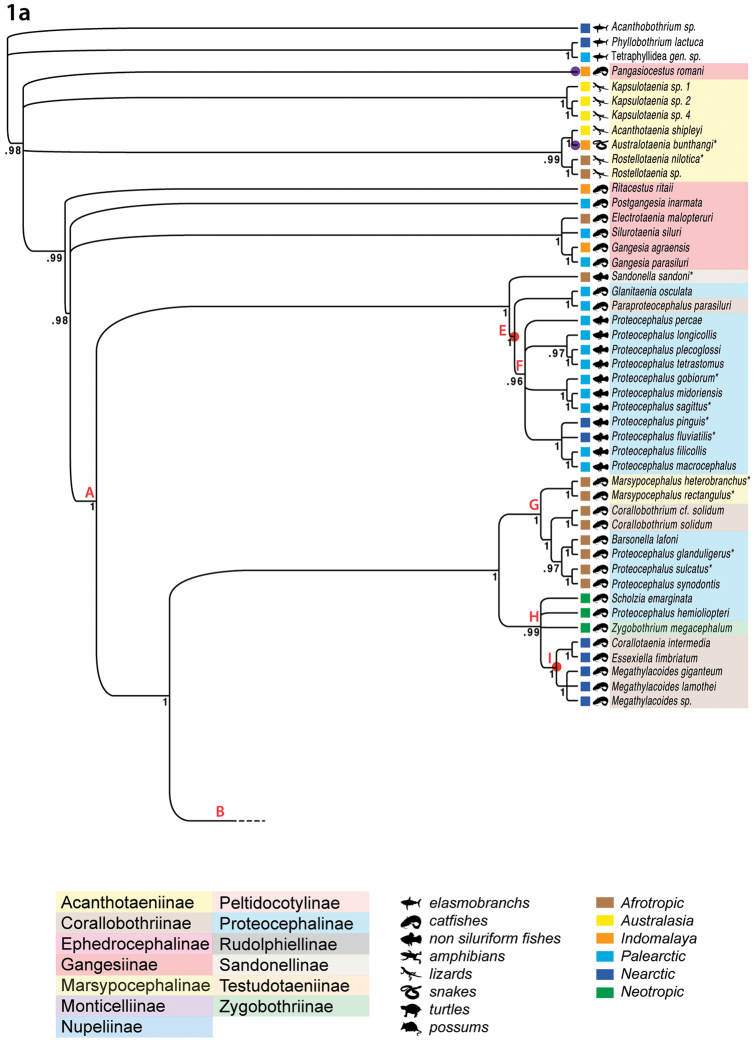

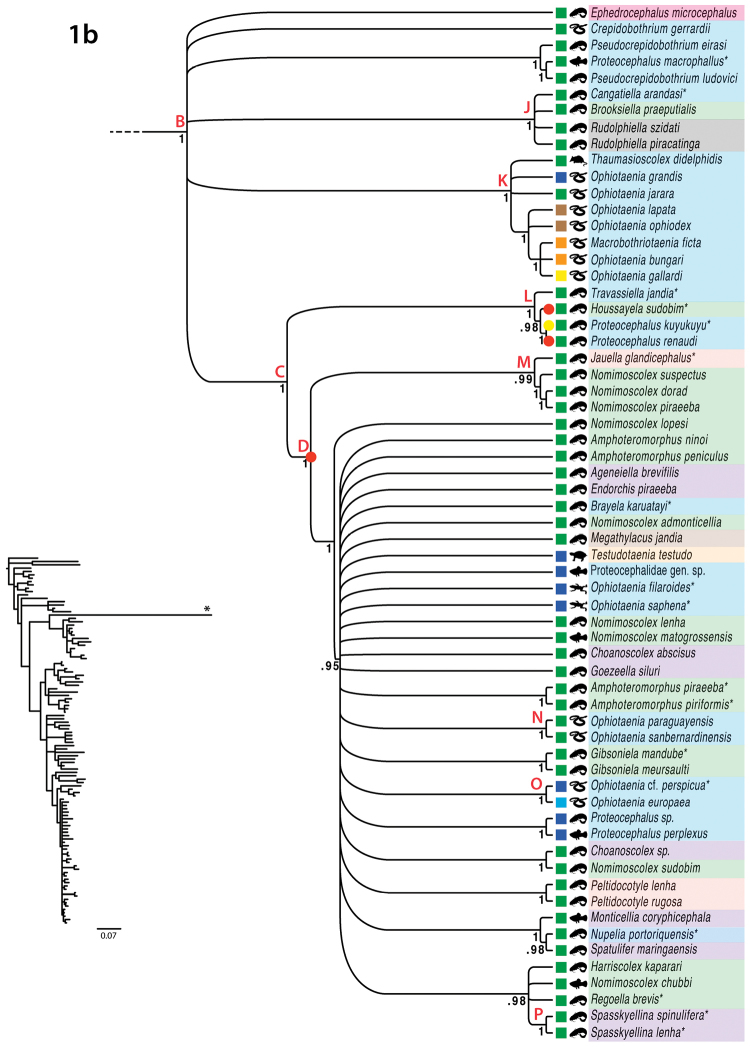
Bayesian inference of partial (domains 1–3) 28S rDNA sequences of a reduced taxon set of proteocephalideans (unstable taxa *Sciadocephalus megalodiscus*, *Vermaia pseudotropii* and *Manaosia bracodemoca* have been removed) performed using MrBayes version 3.2 using the GTR + I + G model of sequence evolution. Two parallel runs were performed for 10,000,000 generations; 4,000,000 generations were discarded as burnin. Branches with posterior probability (pp) support below 95% are collapsed; pp are indicated below branches. Asterisks mark new sequences. Red letters A to P refer to specific nodes discussed in the text. Red circles refer to the acquisition of “Type 2” uterus development; purple circles: acquisition of “intermediate type” uterus development; yellow circle: uterus development unknown (see Discussion). A mute phylogram of the same tree is inserted and the long branch leading to *Sandonella sandoni* is marked with an asterisk.

In an initial BI analysis, several nodes had posterior probabilities (pp) < 0.95, resulting in a tree with only 60 well-supported nodes (see Suppl. material 1: Fig. 1). In order to identify unstable taxa for subsequent exclusion, a leaf stability test was conducted. This revealed *Vermaia pseudotropii* (Verma, 1928), *Sciadocephalus megalodiscus* Diesing, 1850 and *Manaosia bracodemoca* Woodland, 1935 to be the least stable taxa (see Suppl. material 2: Table 1). Curiously, the position of the longest branching taxon, *Sandonella sandoni* (Lynsdale, 1960), was very stable (Fig. 1b inset; Suppl. material 1: Fig. 1, Suppl. material 2: Table 1). The positions of the excluded taxa were as follows: *Vermaia pseudotropii* was in an unresolved position at the base of the tree, *Sciadocephalus megalodiscus* was in an unresolved position in a clade composed of the ingroup taxa to the exclusion of Gangesiinae Mola, 1929 and Acanthotaeniinae Freze, 1963, and *Manaosia bracodemoca* was in an unresolved position in the large subclade of *Clade D* (Suppl. material 1: Fig. 1).

In a subsequent BI analysis, in which the above-mentioned three taxa had been excluded, three nodes had improved support (≥ 0.95 pp), resulting in 63 well-supported nodes in total (Fig. 1a, b). Thus, further topology descriptions are based on the better-supported tree in which nodes of particular interests were labeled *Clades A*–*P* (Fig. 1a, b). Specifically, those better-supported nodes concern the positions of (i) *Postgangesia inarmata* de Chambrier, Al-Kallak & Mariaux, 2003, (ii) *Ritacestus ritaii* (Verma, 1926), and (iii) the sister-group relationship between *Choanoscolex* sp. and *Nomimoscolex sudobim* Woodland, 1935 (Fig. 1a, b; Suppl. material 1: Fig. 1). Thus, the Gangesiinae were shown to be non-monophyletic except for a clade composed of *Electrotaenia malopteruri* (Fritsch, 1886), *Silurotaenia siluri* (Batsch, 1786) and *Gangesia* spp. (Fig. 1a).

The three earliest diverging lineages were formed of *Pangasiocestus romani* Scholz & de Chambrier, 2012 and the Acanthotaeniinae, where the Acanthotaeniinae were possibly non-monophyletic, split into a monophyletic *Kapsulotaenia* Freze, 1965, and a monophyletic assemblage of *Acanthotaenia shipleyi* + *Australotaenia bunthangi* + *Rostellotaenia* spp. (posterior probability = 0.88; not shown), but where all three lineages took an unresolved position at the base of the tree.

The Gangesiinae formed three paraphyletic lineages composed of *Ritacestus ritaii*, *Postgangesia inarmata*, and a clade composed of *Electrotaenia malopteruri*, *Silurotaenia siluri* and *Gangesia* spp. (Fig. 1a), to the exclusion of the remainder of the tree (*Clade A*).

The remainder of the tree (*Clade A*) was structured as follows: The earliest diverging group consisted of *Sandonella sandoni* (Lynsdale, 1960) which parasitizes an ancient osteoglossiform fish in Africa and which formed the sister group to *Clade E*. The latter was composed of two monotypic sister taxa *Glanitaenia* de Chambrier, Zehnder, Vaucher & Mariaux, 2004 (Proteocephalinae) and *Paraproteocephalus* Chen in Dubinina, 1962 (Corallobothriinae), both of which parasitize silurid catfishes in the Palearctic Region. These, in turn, formed the sister group to *Clade F*, which was composed of the *Proteocephalus* aggregate (see de Chambrier et al. 2004c) from Holarctic teleosts, including two newly added species from North America, *Proteocephalus fluviatilis* Bangham, 1925 and *Proteocephalus pinguis* La Rue, 1911.

The next well-supported group structured of *Clade G*, which was exclusively composed of taxa from African siluriforms belonging to three subfamilies (Corallobothriinae, Marsypocephalinae and Proteocephalinae), and which formed the sister group to *Clade H*. The latter was composed of *Scholzia emarginata*, *Proteocephalus hemioliopteri* de Chambrier & Vaucher, 1997 and *Zygobothrium megacephalum* Diesing, 1850, all of which are anatomically similar parasites of the Neotropical catfish *Phractocephalus hemioliopterus* (Bloch & Schneider, 1801), but which are traditionally placed in different subfamilies, and of a monophyletic group of Nearctic proteocephalideans (*Clade I*), all parasitizing channel catfish (Ictaluridae); members of *Clade I* are placed in the Corallobothriinae because they possess a metascolex.

The most derived assemblage, *Clade B*, remained largely unresolved, with five early diverging lineages composed of (i) *Ephedrocephalus microcephalus* Diesing, 1850, (ii) *Crepidobothrium gerrardii* Monticelli, 1900, (iii) a clade of *Pseudocrepidobothrium* spp. + *Proteocephalus macrophallus* (Diesing, 1850), (iv) *Clade J*, composed of *Rudolphiella* spp. + *Cangatiella arandasi* Pavanelli & Machado dos Santos, 1991 + *Brooksiella praeputialis* (Rego, Santos & Silva, 1974), and (v) *Clade K*, composed of *Ophiotaenia* spp., *Macrobothriotaenia ficta* (Meggitt, 1931), all parasites of snakes from various zoogeographical regions, and *Thaumasioscolex didelphidis* Cañeda-Guzmán, de Chambrier & Scholz, 2001, the only proteocephalidean found in possums; (i)–(iv) were exclusively from the Neotropics.

The large polytomy found in *Clade C* was, to a large degree, composed of proteocephalideans parasitizing South American fishes (predominantly siluriforms of the families Pimelodidae, Auchenopteridae and Doradidae). *Clade L* formed the earliest diverging lineage of *Clade C* and was composed of *Travassiella jandia* (Woodland, 1934), *Houssayela sudobim* (Woodland, 1935) and *Proteocephalus kuyukuyu* Woodland, 1935 and *Proteocephalus renaudi* de Chambrier & Vaucher, 1994. The sister group to the large polytomy in *Clade C* was formed of *Clade M*, which included *Jauella glandicephalus* Rego & Pavanelli, 1985, *Nomimoscolex suspectus* Zehnder, de Chambrier, Vaucher & Mariaux, 2000, *Nomimoscolex dorad* (Woodland, 1935) and *Nomimoscolex piraeeba* Woodland, 1934. The remainder of *Clade C* formed largely a comb which comprised, amongst others, *Testudotaenia testudo* (Magath, 1924), a parasite of North American soft-shelled turtles and bowfin (*Amia calva*), a clade of *Proteocephalus* sp. and *Proteocephalus perlexus* La Rue, 1911, parasitizing North American catfish and bowfins respectively, two distinct clades of *Ophiotaenia* La Rue, 1911, *Clade N* (parasites of South American snakes) and *Clade O* (parasites of European and Nearctic snakes), and two unresolved *Ophiotaenia* species, *Ophiotaenia filaroides* La Rue, 1909 and *Ophiotaenia saphena* Osler, 1931, parasitizing North American salamanders and frogs, respectively.

The possible monophyly of 17 proteocephalidean genera could be examined, at least preliminarily, because two or more species of these genera were included in our analyses (numerous proteocephalidean genera are monotypic or species-poor). According to the current taxon sampling, the following genera, listed alphabetically, appeared monophyletic (the numbers in parentheses indicate the total number of species sequenced and the number of distinct lineages in which species of a given genus appeared): *Corallobothrium* Fritsch, 1886 (2/1), *Gangesia* Woodland, 1924 (2/1), *Gibsoniela* Rego, 1984 (2/1), *Kapsulotaenia* Freze, 1965 (3/1), *Marsypocephalus* Wedl, 1861 (2/1), *Megathylacoides* Jones, Kerley & Sneed, 1956 (3/1), *Peltidocotyle* Diesing, 1850 (2/1), *Proteocephalus* aggregate (11/1), *Rostellotaenia* Freze, 1963 (2/1) and *Spasskyellina* Freze, 1965 (2/1) (see discussion below for the latter). The monophyly of *Rudolphiella* Fuhrmann, 1916 (2/1) was not rejected by these results. In contrast, *Pseudocrepidobothrium* Rego & Ivanov, 2001 (2/2) is paraphyletic and the genera *Amphoteromorphus* Diesing, 1850 (4/3), *Choanoscolex* La Rue, 1911 (2/2), *Nomimoscolex* Woodland, 1934 (9/7), *Ophiotaenia* (12/10) and *Proteocephalus* (20/7) appeared to be polyphyletic based on their current classification.

### Morphological analysis

**Figure 2.**
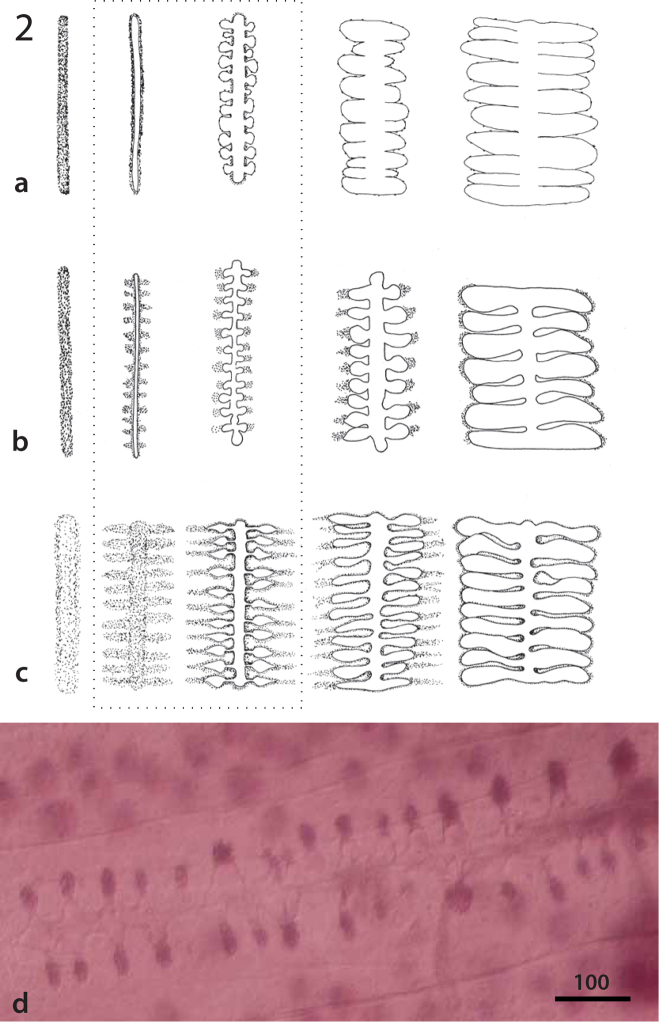
Schematic representation of proteocephalidean uterus development (**a–c**). The uterus observed in early immature, premature, mature, pregravid and gravid proglottids is represented from left to right. The major differences are observed in premature and mature proglottids (dotted line): **a** and **c** Development of Type 1 and 2, respectively (de Chambrier et al. 2004c) **b** Development of an “intermediate type” as observed in *Pangasiocestus* and *Australotaenia* (this paper) **d** Typical “intermediate type” uterus in a mature proglottid of *Australotaenia bunthangi* de Chambrier & Scholz, 2012 (holotype, MHNG-PLAT-75447). Scale in micrometers.

At the morphological level, the ovary to proglottid surface ratio ranged between 2.0% in *Ophiotaenia grandis* La Rue, 1911 to 20.8% in *Zygobothrium megacephalum* (Table 1). Examination of new whole mounts also identified a novel form of the uterine development in addition to those described by de Chambrier et al. (2004c). This development is characterized as follows: in immature proglottids, the uterine stem forms an elongated concentration of chromophilic cells; in premature proglottids the chromophilic cells concentrate in areas where lateral uterine extensions will develop; in mature proglottids, a tubular uterine stem appears and develops small thin-walled lateral diverticula topped with a conspicuous concentration of numerous intensely stained cells; in pregravid and gravid proglottids, the lateral diverticula grow and eventually occupy most of the proglottid width (Fig. 2b, d). We call this development “intermediate type”.

## Discussion

Since the publications of de Chambrier et al. (2004c) and Hypša et al. (2005), no attempt has been made to unravel the phylogenetic structure of proteocephalideans. Two immediate observations can be made when comparing our results to the de Chambrier et al. (2004c) tree: (1) an overall better resolution is achieved with the increased taxon sampling; and (2) all clades that were supported in de Chambrier et al. (2004c) remain so in these results. However, a number of differences can also be noted as discussed below.

### Early diverging lineages – Acanthotaeniinae and Gangesiinae

In both de Chambrier et al. (2004c) and the present study, the Gangesiinae from Siluriformes, mostly in Indomalaya and Palearctic (but with one species in Afrotropics), and Acanthotaeniinae from reptiles in Australasia, Afrotropic and Indomalaya are early diverging lineages. However, their order is now reversed with the Acanthotaeniinae, together with *Pangasiocestus romani* (Gangesiinae), taking the earliest diverging position. Thus, the present results suggest either the paraphyly of the subfamily or the necessity to handle *Pangasiocestus* Scholz & de Chambrier, 2012 as an independent lineage. This monotypic genus was initially placed in the Gangesiinae based on its scolex morphology, which is characterized by a large rostellum-like apical organ. However, it differs from all gangesiine in a number of morphological characteristics. These include the peculiar, rosette-like scolex with a large, discoidal apical organ devoid of hooks; a very weakly-developed inner longitudinal musculature, which does not form bundles (unlike those of other gangesiine genera, which form numerous bundles of muscle fibers; see Scholz et al. 1999, de Chambrier et al. 2003, de Chambrier et al. 2004b, Ash et al. 2012 for more details); and the variable size of testes, which are considerably smaller and denser in the lateral than in the median field. These morphological features support the separation of *Pangasiocestus* from the Gangesiinae, as shown by our genetic analysis, despite the superficial resemblance of its scolex with that of other gangesiine cestodes.

It should also be noted that, together with *Australotaenia* de Chambrier & de Chambrier, 2010, *Pangasiocestus* has a particular, intermediate development of the uterus (see below), that contrasts that of all other Gangesiinae and Acanthotaeniinae, which have a Type 1 development of the uterus. *Pangasiocestus romani* was found in a catfish in Cambodia, and species of *Australotaenia* are distributed in Australia and Indomalaya, which would suggest an Old World origin for proteocephalideans. This scenario is consistent with the results of de Chambrier et al. (2004c) and contradicts the hypothesis of Brooks (1978), who favored a South American origin of the group.

### The *Proteocephalus* aggregate and the enigmatic *Sandonella* and *Sciadocephalus*

The position of *Sandonella* Khalil, 1960 as a separate long-branching lineage, as already observed by de Chambrier et al. (2008), was confirmed in the present study. *Sandonella* formed the sister group to the strongly supported *Clade E*, which is composed of species of the *Proteocephalus* aggregate (*Clade F*) that are parasites of teleosts in the Holarctic Region, and monotypic genera *Glanitaenia* and *Paraproteocephalus*, which are parasites of silurid catfish in the Palearctic Region. The members of the *Proteocephalus* aggregate (= *Proteocephalus* sensu stricto) will retain the generic name since this clade undoubtedly includes *Proteocephalus ambiguus* (Dujardin, 1845), the type species of *Proteocephalus*, as shown by Scholz et al. (2007). The addition of two *Proteocephalus* species of Nearctic origin [*Proteocephalus fluviatilis* from centrarchids (Perciformes) and *Proteocephalus pinguis* from pikes (Esociformes)] to the dataset revealed their affinity with the *Proteocephalus* aggregate. This close phylogenetic relationship of the Palearctic and Nearctic taxa analyzed is in accordance with their similar morphology (Freze 1965, Scholz and Hanzelová 1998). The diversity of hosts in *Clade E* is surprising when compared to other subgroups of proteocephalideans that generally diversify in discrete groups of catfish. In this case a Holactic radiation of these cestodes in multiple groups of fishes has occurred.

*Sandonella sandoni* was placed in a new genus and subfamily, Sandonellinae, mostly because of the characteristic posterior position of its vitellarium, which is unique among proteocephalideans and somewhat resembles that of the Cyclophyllidea in being formed by two compact, yet deeply lobulated postovarian masses near the posterior margin of the proglottids (Khalil 1960, see also fig. 6 in de Chambrier et al. 2008). Bâ and Marchand (1994) observed the unique structure of *Sandonella sandoni* spermatozoa (with a single axoneme) and de Chambrier et al. (2008) reported its widespread presence in *Heterotis niloticus* (Cuvier, 1829) throughout Africa and described additional original morphological characters such as a scolex with a highly modified apical structure formed by 4 muscular retractile lappets, a dilated, vesicle-like proximal part of the external sperm duct, a unique morphology of the uterus, and a complex proglottization with mixed smaller and larger (wider) proglottids. Despite these peculiarities, as well as its derived 28S sequence, the position of *Sandonella sandoni* as a sister group of Holarctic Proteocephalinae was established by de Chambrier et al. (2008) and is not questioned by these results. The presence of this relatively derived parasite in a basal fish lineage (Osteoglossiformes) is further evidence that the evolution of proteocephalideans does not closely match that of their hosts. It should be noted though that the phylogenetic position of this taxon has not yet been tested in more global cestode phylogenies (i.e. Waeschenbach et al. 2012, Caira et al. 2014).

*Sciadocephalus megalodiscus* parasitizing *Cichla monoculus* Agassiz, 1831 (Perciformes) in the Neotropical region and described by Diesing (1850) is another enigmatic taxon. In its redescription Rego et al. (1999) noted several peculiar morphological features, such as an umbrella-shaped metascolex, a uterus rapidly resolving into capsules, and a musculature with numerous isolated longitudinal fibers, and placed the species in the Corallobothriinae based on the presence of a metascolex [which is, however, a homoplastic character (Scholz et al. 2013)] and the medullary position of the genital organs. In our initial evaluation, this taxon appeared as the earliest diverging lineage of *Clade A* (see Suppl. material 1: Fig. 1) but it has also been identified as one of the three least stable taxa in the analysis and had therefore been excluded from further analyses. Nevertheless, this possible distinct position of the species among proteocephalideans, supported by its combination of peculiar morphological characteristics, might justify its future placement in a separate, higher taxonomic group.

### African fish proteocephalideans

Our considerably enlarged dataset of fish proteocephalideans from Africa covers most of their diversity and includes all genera reported from the Afrotropical Region. It revealed that all but one species (the gangesiine *Electrotaenia malopteruri* – see above) from African siluriform fish form a well-supported, relatively basal *Clade* G. This is one of the most important novelties of the present study: species placed in three subfamilies are phylogenetically closely related despite important morphological differences. These are: i) the Corallobothriinae (two species of *Corallobothrium* including its type species from malapterurid electric catfish) characterized mainly by a well-developed metascolex and medullary testes; ii) the Marsypocephalinae (two species from clariids) with a simple scolex and cortical testes; and iii) the Proteocephalinae (three *Proteocephalus* species from clariid, claroteid and mochokid catfish, and *Barsonella lafoni* de Chambrier, Scholz, Beletew & Mariaux, 2009 from *Clarias* spp.), with a relatively simple scolex and medullary testes (de Chambrier et al. 2009b). This grouping of taxa with markedly different scoleces as well as conspicuously distinct position of the testes (medullary versus cortical) is further evidence that morphological characteristics related to the scolex and internal topology of genital organs are homoplastic and should be interpreted with great caution. A similar situation was demonstrated in *Macrobothriotaenia ficta*, a snake parasite from Indomalaya, which possesses a tetraphyllidean-like scolex: it is closely related to species of *Ophiotaenia* with a simple scolex (Scholz et al. 2013; see also *Clade K*), but less so with *Thaumasioscolex didephidis* despite having a very similar scolex morphology. The new results also indicate that zoogeography and host associations may have played a much more important role in the evolutionary history of proteocephalidean cestodes than previously thought (Freze 1965, Rego et al. 1998).

### Parasites of the Neotropical pimelodid catfish *Phractocephalus hemioliopterus*

Neotropical catfish, in particular pimelodids, harbour the highest number of species (and genera) of proteocephalidean cestodes. However, these parasites do not form a monophyletic assemblage, even though most of them belong to our most derived clade with unresolved internal relationships (see also Zehnder and Mariaux 1999, de Chambrier et al. 2004c). The current study confirmed the polyphyly of these cestodes, including the markedly distant position of three species from the pimelodid catfish *Phractocephalus hemioliopterus* (*Clade H*) from the remaining cestodes parasitizing other siluriforms from South America, as first observed in a much smaller dataset by Hypša et al. (2005).

As many as six species reported from *Phractocephalus hemioliopterus* were included in our analyses. Three of them, namely *Proteocephalus hemioliopteri*, *Scholzia emarginata* (both Proteocephalinae) and *Zygobothrium megacephalum* (Zygobothriinae), differ markedly from each other in their scolex morphology (see de Chambrier et al. 2005), yet form a well-supported lineage (*Clade H*) together with Nearctic “corallobothriines” (*Clade I*). Their phylogenetic position is, thus, more basal and distant from that of other proteocephalideans parasitizing Neotropical teleosts.

The remaining three taxa that parasitize *Phractocephalus hemioliopterus*, i.e. two species of *Pseudocrepidobothrium* (Proteocephalinae) and *Ephedrocephalus microcephalus* Diesing, 1850 (Ephedrocephalinae) group in an unresolved position towards the base of the South American radiation. This suggests possible independent colonizations of this host. The basal position of these parasites is in accordance with the fact that *Phractocephalus hemioliopterus* is one of the most ancient pimelodids, as suggested by fossil records dating from Middle to Late Miocene (Lundberg and Littmann 2003).

Our data do not enable any reliable assessment regarding a possible host-parasite coevolution, especially in the case of pimelodid catfishes and their Neotropical proteocephalideans. A comparison of the interrelationships of the Pimelodidae based on robust morphological and molecular evidence (Lundberg et al. 2011 and references therein) with the present data does not reveal any obvious pattern of possible co-evolutionary history. In fact, cestodes from closely related pimelodids such as species of *Pseudoplatystoma* Bleeker, 1862 and *Sorubimichthys planiceps* (Spix & Agassiz, 1829) are unrelated and belong to distant lineages (Table 1 and Fig. 1a, b).

### Nearctic “corallobothriines” from channel catfishes (Ictaluridae)

Nearctic species from channel catfish form a well-supported, monophyletic lineage (*Clade I*) composed of species of three genera, *Essexiella* Scholz, de Chambrier, Mariaux & Kuchta, 2011, *Megathylacoides* and *Corallotaenia* Freze, 1965. However, the Nearctic genera, conventionally placed in the Corallobothriinae because they possess a metascolex, are not closely related to the monotypic *Corallobothrium* from the electric catfish, *Malapterurus electricus* Gmelin, 1789, in Africa and their morphological resemblance is probably a result of convergent evolution (Scholz et al. 2011). In fact, the subfamily Corallobothriinae groups species of unrelated genera (African *Corallobothrium* in *Clade G*, three Nearctic genera in *Clade I*, Japanese *Paraproteocephalus* in *Clade E* and Neotropical *Megathylacus* Woodland, 1934 in *Clade D* – Fig. 1a, b) that share apparently homoplasious morphological characteristics, i.e. a well-developed metascolex and a medullary position of genital organs as described above (Freze 1965, Rego 1994, Rosas-Valdez et al. 2004).

As a consequence, a new taxon should be proposed to accommodate Nearctic channel catfish proteocephalideans, which are apparently unrelated either to the true corallobothriines (in fact now represented by *Corallobothrium solidum* and a species to be described, both from Africa) or to the various other proteocephalideans from freshwater teleosts in North America that are distributed throughout the phylogenetic tree (*Clades F* and *D* – see Fig. 1a, b). Similarly, the position of *Paraproteocephalus* within the Corallobothriinae will need to be reconsidered. This placement is likely to be due to convergences in scolex shape, and the genus should be placed in the Proteocephaliinae.

### Cosmopolitan reptilian proteocephalideans

The distribution of proteocephalideans in snakes is particularly interesting. Multiple colonizations of reptiles, as already suggested by de Chambrier et al. (2004c), are confirmed here and at least three main events (see *Clades K*, *N* and *O*) are shown in this study (besides the case of *Australotaenia*). In each case, cestodes of snakes appear to be related to various proteocephalideans of Neotropical catfishes and other teleosts (Fig. 1a, b). The most interesting novel insight from our study in this context is the strong support found for *Clade K*, composed almost exclusively of parasites from snakes (Viperidae, Elapidae, Lamprophiidae and Xenopeltidae) throughout the world (with the exception of Palearctic) and the unique switch to a mammalian host (*Didelphidis marsupialis* L., 1758) in the northernmost Neotropical Region in the case of *Thaumasioscolex didelphidis*. Colubridae are notably absent from this host list. This grouping of rather derived snake parasites cannot be unambiguously explained by our data and may either be the sign of a relatively recent colonization of unrelated groups in all continents or a trace of a very ancient colonization of snakes. Even though all these species belong to the Proteocephalinae because of the medullary position of their genital organs and the absence of a metascolex, they actually differ markedly from each other, especially in their scolex morphology, and were placed in three separate genera (Freze 1965, de Chambrier 1989a, de Chambrier 1989b, Rego 1994, Cañeda-Guzmán et al. 2001, Scholz et al. 2013). Two of these (*Macrobothriotaenia* Freze, 1965 and *Thaumasioscolex*) are essentially characterized by peculiar scoleces. The position of *Crepidobothrium gerrardii* (Monticelli, 1900), a parasite of Boidae that is also characterized by a distinctive scolex, is not fully resolved but is possibly unrelated to this radiation.

Species of *Ophiotaenia* in colubrids from Holarctic (2 species – *Clade O*), Neotropical dipsadids (2 species – *Clade N*), and Nearctic amphibians are possibly unrelated and appear within a polytomy composed of numerous lineages of Neotropical fish proteocephalideans. They are morphologically uniform and do not differ significantly from the other species of *Ophiotaenia* in *Clade K*, as all of them have a similar scolex and strobilar morphology, including relative ovary size (see de Chambrier et al. 2012 and Table 1). However, members of the larger radiation (*Clade K*) have a Type 1 uterus whereas those in the other clades have a Type 2 uterus. Consequently, and as suspected (Ammann and de Chambrier 2008), it is clear that *Ophiotaenia* is a composite genus and this name should be restricted to species of *Clade O*, which includes the type species *Ophiotaenia perspicua* La Rue, 1911 from Neartic colubrids. Species in *Clade O* have proportionally larger ovaries than those in the remaining species of “*Ophiotaenia*” (*Clades K*, *N*), which will need to be allocated to other (new) genera.

### “Neotropical fish” superclade

In addition to the above-mentioned “reptilian” lineages, our derived *Clade B* is composed of a number of Neotropical parasites of catfishes and a few other teleosts, where the highest species richness can be found in the Pimelodidae (Siluriformes) (de Chambrier and Vaucher 1999, Rego et al. 1999). A few parasites from amphibians and turtles, as well as *Proteocephalus perplexus* La Rue, 1911 from bowfin (*Amia calva* L., 1766), also belong to this large polytomy. de Chambrier et al. (2009a) showed that *Testudotaenia* Freze, 1965 of the monotypic subfamily Testudotaeniinae was part of a North American clade of proteocephalid parasites of fishes despite its distinctive morphology. These results do not contradict this hypothesis although *Testudotaenia*’s closest relatives cannot be inferred from the present tree.

Despite our enlarged sample size, the present study did not resolve the relationships of most Neotropical proteocephalideans from teleosts, and in this respect does not significantly improve the results of Zehnder and Mariaux (1999), de Chambrier et al. (2004c) or Hypša et al. (2005). Still, some nodes are now well supported, e.g., species of *Brooksiella* Rego, Chubb & Pavanelli, 1999, *Rudolphiella* and *Cangatiella* Pavanelli & Machado dos Santos, 1991 (*Clade J*), species of *Travassiella* Rego & Pavanelli, 1987, *Houssayela* Rego, 1987 and two species of “*Proteocephalus*” (*Clade L*), and three species of the largely polyphyletic *Nomimoscolex*, including *Nomimoscolex piraeeba* (type species), together with *Jauella glandicephalus* (*Clade M*). However, these well-supported lineages are composed of species with dissimilar morphologies and often belong to different subfamilies (as many as three in *Clade J*). In addition, they parasitize fish of different genera, families or even orders, which makes it impossible to define them logically for now.

Other molecular markers, possibly large mtDNA fragments, as used by Waeschenbach et al. (2012), are obviously needed if the internal phylogenetic structure of the derived *Clade B* is to be unravelled, although the possibility that this node represents a hard-polytomy should also be considered. A similar situation, i.e. support for some of the internal nodes but a lack of support for the major lineages, was observed for the Caryophyllidea, another order of fish tapeworms, despite the use of several nuclear and mitochondrial markers. These commonly employed molecular markers did not contain sufficient phylogenetic signal due to substitution saturation (Brabec et al. 2012).

Catfishes (order Siluriformes) represent one of the key host groups for proteocephalidean cestodes, but there is no obvious coevolutionary pattern between them. This lack of closer host-associations at a higher taxonomic level is not surprising because catfishes form an extraordinarily diverse group of teleosts with over 3,000 valid recognized species (Eschmeyer et al. 2004). The interrelationships of large groups in the Siluroidei, which comprises almost all catfish hosts of proteocephalideans, including the Neotropical pimelodids and heptapterids (Pimelodoidea) and African taxa (“Big Africa” clade with cestode-hosting families Mochokidae, Malapteruridae, and Auchenoglanidae and phylogenetically distant Clariidae) are poorly resolved (Sullivan et al. 2006). Molecular data suggest an ancient siluriform presence, if not origin, in South America, but phylogenies inferred from *rag* gene sequences did not identify any African-South American catfish clade (Sullivan et al. 2006).

### Monophyly/polyphyly of proteocephalidean genera

Even though 10 genera (see above) appeared to form monophyletic assemblages, all but one (*Proteocephalus* aggregate) were represented by a very low number of species (2–3), and the validity of some of them may still have to be reconsidered when a denser sampling is available. In contrast, all species-rich genera with at least nine species analyzed (*Nomimoscolex*, *Ophiotaenia* and *Proteocephalus* sensu lato), as well as *Amphoteromorphus* (4 species), appeared to be polyphyletic and are distributed across numerous lineages, even though their morphology and host-associations are quite similar.

A situation comparable to that of *Proteocephalus* (species of this genus belong to at least 7 distinct lineages – Fig. 1a, b) starts to emerge in *Nomimoscolex*. As previously noted by Zehnder et al. (2000), our *Nomimoscolex* samples are distributed across several distinct lineages in *Clade D*. The type species *Nomimoscolex piraeeba*, belonging to the well-supported *Clade M*, and all *Nomimoscolex* loosely grouped across other lineages in *Clade D* will ultimately have to be placed in other genera. At this point, however, objective morphological characters are still lacking to recognize these worms.

This work also confirms the polyphyly of *Monticellia* La Rue, 1911 in its present form with *Monticellia spinulifera* Woodland, 1935 and *Monticellia lenha* Woodland, 1933 found in siluriforms forming well-supported *Clade P*, which is distantly related to the type species of the genus, *Monticellia coryphicephala* (Monticelli, 1891) from characids. The two former species belong to *Monticellia* since de Chambrier and Vaucher (1999) synonymised *Spasskyellina* Freze, 1965 with *Monticellia*. *Spasskyellina* was later considered as valid by de Chambrier et al. (2006), without considering the 1999 work, thus generating confusion about the status of the genus. Given the obvious morphological support that confirms our molecular results, we propose splitting *Monticellia* in order to reflect this situation and to formally resurrect here the genus *Spasskyellina*, that was erected in 1965 by Freze (Freze 1965) for those taxa possessing gladiate spinitriches (de Chambrier and Scholz 2008, Chervy 2009) on margins of their suckers, i.e. *Spasskyellina lenha* (Woodland, 1933) Freze, 1965 (type species) and *Spasskyellina spinulifera* (Woodland, 1935a) Freze, 1965. They are presented under this name in Fig. 1b. Additionally, *Spasskyellina mandi* Pavanelli & Takemoto, 1996 is confirmed in this revalidated genus because of its obviously similar morphology, contrary to previous observations (Pavanelli and Takemoto 1996, de Chambrier and Vaucher 1999). Since molecular data for other species of *Monticellia* are not available, they are provisionally kept in that genus.

### Evolution of morphological characters

**Figure 3.**
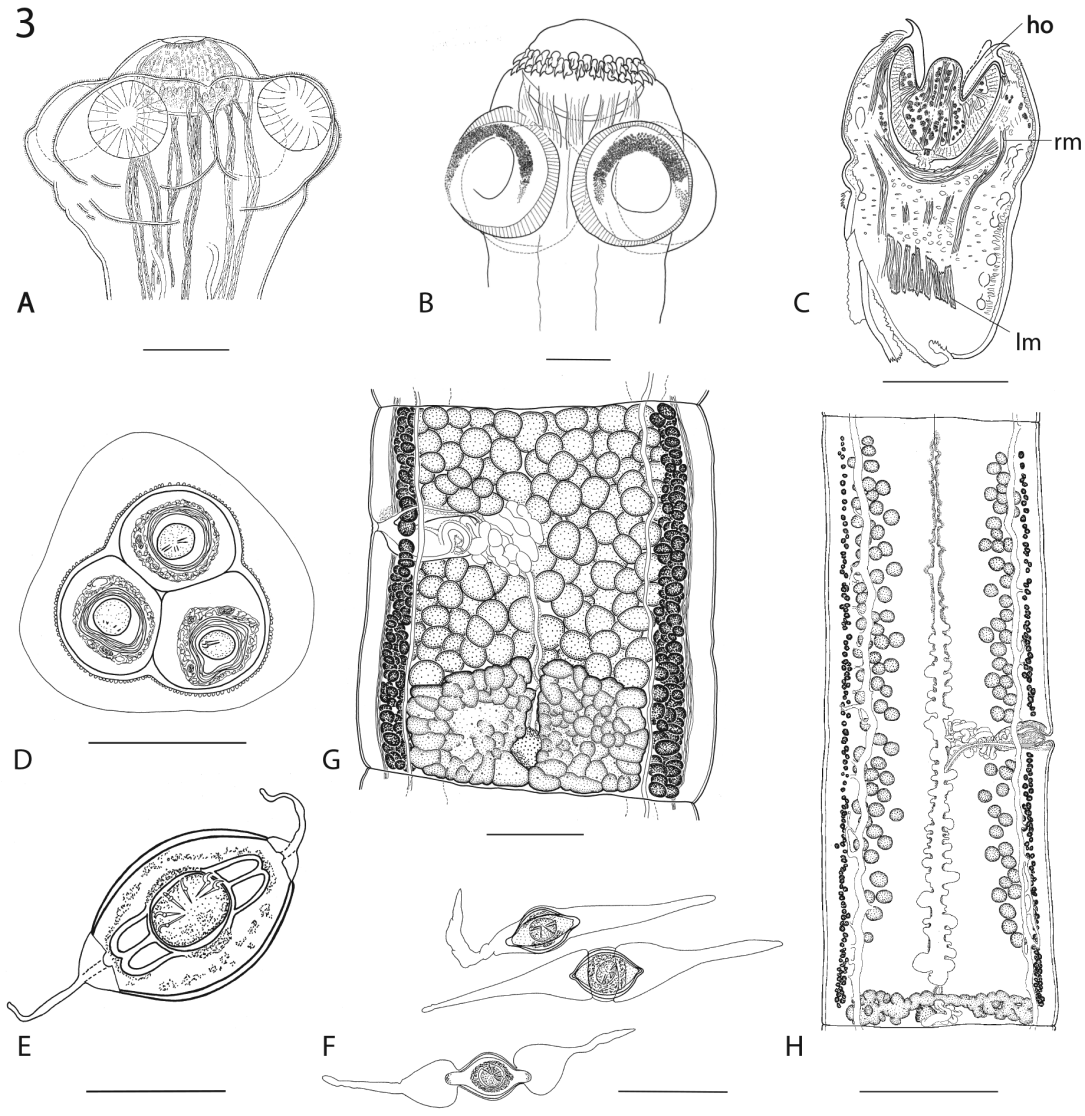
**A–C** Scoleces with rostellum-like organs and retractor muscles. **A** Without hooks. *Ritacestus ritaii* (Verma, 1926) (modified from de Chambrier et al. 2011) **B** With hooks. *Gangesia bengalensis* (Southwell, 1913) (modified from Ash et al. 2012) **C** Partly-invaginated. Sagittal section, ho: hooks; rm: retractor muscles; lm; longitudinal muscles. *Vermaia pseudotropii* (Verma, 1928) (modified from Ash et al. 2010) **D–F** Egg modifications **D** Egg cluster in a capsule. *Vandiermenia beveridgei* (de Chambrier & de Chambrier, 2010) (modified from de Chambrier and de Chambrier 2010) **E** Egg with two polar projections. *Brooksiella praeputialis* (Rego, Santos & Silva, 1974) (modified from de Chambrier et al. 2004a) **F** Eggs with two polar projections. *Rudolphiella* spp. from *Calophysus macropterus* (two eggs above) and *Megalonema platanum*, respectively (modified from Gil de Pertierra and de Chambrier 2000) **G–H** Ovary size **G** Relatively large ovary (16.4% proglottid surface) in *Gangesia agraensis* Verma, 1928 (modified from Ash et al. 2012) **H** Relatively small ovary in *Ophiotaenia lapata* Rambeloson, Ranaivoson & de Chambrier (2012) (2.8% of proglottid surface) (modified from Rambeloson et al. 2012). Scale-bars: **A, B, C** = 100 µm; **D, E** = 20 µm; **F** = 50 µm; **G** = 200 µm; **H** = 500 µm.

Regarding the evolution of morphological characters, the most obvious and evolutionarily important observation derived from Fig. 1a, b is the presence of a rostellar apparatus with retractor muscles in all the basal taxa. Such structures (Fig. 3A–C), although with some variation, are characteristic of all Acanthotaeniinae and Gangesiinae and are lost in all more derived Proteocephalidae (*Clade A*) without exception. Although apical structures are present in some other members of the order such as in the *Proteocephalus* aggregate from the Holarctic (see Scholz et al. 1998), *Proteocephalus sophiae* de Chambrier & Rego, 1994 from South America, *Proteocephalus glanduligerus* (Janicki, 1928) from Africa, *Jauella* Rego & Pavanelli, 1985 or *Nomimoscolex* sensu stricto as defined by Zehnder et al. (2000) (*Clade M*), these are very different, especially because they lack a supporting muscular apparatus (retractors) (de Chambrier and Rego 1994, de Chambrier and Vaucher 1999, Scholz et al. 2009). This kind of functional simplification, in this case due to the loss of apical attachment structures, is known from other cestode groups and has appeared repeatedly, for example in a number of derived cyclophyllidean genera (Jones et al. 1994), even though these structures are unlikely to be homologous.

The development of the uterus seems to represent one of the key features that reflects the evolution of proteocephalideans and characterizes their major lineages. The evolution of uterine structure as described in de Chambrier et al. (2004c) is essentially supported in the present analysis although with some added complexity. Both putative acquisitions of Type 2 uterine development observed by these authors are observed in our extended analysis (see red circles in *Clade E* and *D*) but the inclusion of new taxa revealed a third instance of transition of this character in *Clade I* in a well-supported group of Nearctic Corallobothriinae. Furthermore, the situation for taxa belonging to *Clade L* is unclear with two of them harbouring a Type 2 uterus, one a Type 1 uterus (*Travassiella jandia*) and one with missing information (gravid proglottids of *Proteocephalus kuyukuyu* have never been found).

Two basal taxa belonging to Acanthotaeniinae and Gangesiinae show a different, as yet undescribed, form of uterus development that we call “intermediate type” (see purple circles on Fig. 1a). This development differs from Type 1 development by the presence of chromophilic cells at points of origin of the lateral extensions of the uterus before the lateral stems are visible. It differs from Type 2 development in an early appearance of the main tubular uterus axis (Fig. 2). Assuming that the “intermediate type” might be a transitional stage between both uterus development types, a possible interpretation of this observation would be that a general trend toward the acquisition of Type 2 uterus development exists throughout the proteocephalidean diversity.

New morphological characters that are potentially useful for proteocephalidean taxonomy are notoriously difficult to define. However, Ammann and de Chambrier (2008) observed differences in the relative surface area of the ovary in relation to the total surface of the proglottids (see Fig. 3G–H). In their study, this ratio was on average five times lower in 27 species of *Ophiotaenia* from snakes in the New World compared to Palearctic members of the *Proteocephalus* aggregate from teleosts. More recently, de Chambrier et al. (2012) compared 66 of the nominal species of *Ophiotaenia* from Old and New World reptilian hosts with 69 species of *Proteocephalus* from freshwater teleosts. They noted that the ovaries of species parasitic in non-Palearctic snakes are proportionally smaller than those in species of *Proteocephalus* parasitic in teleost fishes from all over the world and also considerably smaller than that of congeneric species from European hosts.

In the present study, data on the relative size of the ovary are provided for all taxa analyzed (see Table 1). Results from two former studies (Ammann and de Chambrier 2008, de Chambrier et al. 2012) are verified here in the context of a larger dataset covering more genera and subfamilies. We can conclude that the ratio of the ovary surface to the proglottid surface in mature proglottids largely corresponds to major host groups and thus represents a promising character of possible phylogenetic importance that should be routinely reported in future descriptions or redescriptions of proteocephalidean taxa (for methodology of taking this ratio – see de Chambrier et al. 2012). However, patterns in the relative size of the ovary of species from different host groups discussed above are not universal and notable exceptions exist. For example, the smallest known ovary is found in *Margaritaella gracilis* Arredondo & Gil de Pertierra, 2012 from the catfish *Callichthys callichthys* (L., 1758) (ratio of 0.6–1.8%; Arredondo and Gil de Pertierra 2012) and not in a species from snakes.

Characters related to eggs and their morphology have been shown to be important in the systematics of proteocephalidean cestodes (Gil de Pertierra and de Chambrier 2000, Scholz and de Chambrier 2003, de Chambrier et al. 2005, de Chambrier 2006, de Chambrier and de Chambrier 2010, Scholz et al. 2011) but have generally been underexploited and remain poorly known for many species. Here, they allow the characterization of a well-supported node grouping species of *Rudolphiella*, *Brooksiella* and *Cangatiella* (*Clade J*), because all these taxa possess very typical eggs with polar extensions (Fig. 3E, F). To our knowledge, no other proteocephalidean shows such egg characteristics and thus the presence of polar extensions can be considered as a synapomorphy that defines this group. Furthermore, species in these genera all present a ventral vitellarium and *Brooksiella* and all species of *Rudolphiella* (but not *Cangatiella*) have a folliculate ovary and a metascolex (Gil de Pertierra and Viozzi 1999, de Chambrier et al. 2004b). These morphological characteristics seem to strongly support this clade.

Another kind of egg (in capsules) (Fig. 3D) is found in the basal Australasian *Kapsulotaenia* parasites of varanids and is also known in *Vandiermenia* de Chambrier & de Chambrier, 2010 and some “*Ophiotaenia*” of Australian snakes. In the Neotropics a similar evolution of eggs (in groups of 4–6) is known in *Thaumasioscolex*, the single known proteocephalidean of marsupials. The phylogenetic value of this character remains presently doubtful as some of these worms belong to isolated clades (Scholz et al. 2013). It may however represent an interesting convergent adaption in proteocephalidean with terrestrial life cycle, although it curiously did not seem to have appeared outside of the Autralasian (and maybe Neotropical) region despite the presence of terrestrial proteocephalideans in other areas.

Unfortunately, most lineages revealed in the present study lack such obvious synapomorphies due to a high degree of homoplasy across numerous morphological characters previously used for distinguishing individual genera and subfamilies, such as scolex morphology and the position of reproductive organs in relation to the inner longitudinal musculature (Rego 1994, 1999). Thus, the delineation of many taxonomic groups using morphological features remains currently impossible.

## Conclusions

This study is based on the most representative molecular dataset of proteocephalidean taxa ever sampled (33% of all valid species, almost 80% of genera and all extant subfamilies). However, some groups are still under-represented, mainly because of the difficulties in obtaining fresh samples, either due to their low prevalence and the protection or rare occurrence of their hosts. Probably the most serious gap in our dataset is the small number (two species) of proteocephalideans parasitizing amphibians (frogs and salamanders). These are usually extremely rare, with less than 1% of host infected (de Chambrier et al. 2006, Marsella and de Chambrier 2008). Similarly, none of the four species of *Ophiotaenia* from lizards (excluding *Varanus* spp.) were available for this analysis. In contrast, our geographical coverage was rather comprehensive thanks to the intensive sampling effort during the last decades. This considerably enlarged dataset has helped us to better characterize several lineages, but the relationships of many taxa, especially those in the most derived *Clade B*, largely comprising parasites of catfishes in the Neotropical Region, remain largely unresolved.

The evolutionary history of the order has been apparently much more complicated than one would expect, considering a relatively small number (about 315) of extant species. Although we did not formally examine the host-parasite coevolution of proteocephalideans here, our tree strongly suggests the occurrence of several colonization events of poikilothermic vertebrates as well as repeated colonization of the principal zoogeographical regions with the most recent, and probably explosive, radiation in Neotropical teleosts, especially pimelodid catfishes.

Based on 28S rDNA sequences, these results support several new insights into the evolution of proteocephalideans. Unfortunately, they also cast a number of doubts on our present understanding of the classifications within this group: most recognized subfamily-level taxa are, at best, only partially supported. A notable consequence is that scolex morphology and the position of internal organs (testes, uterus and vitelline follicles in relation to the inner longitudinal musculature) should be considered with caution when used for higher-level taxonomy, i.e. to distinguish genera and subfamilies. Clearly a complete taxonomical reorganization of the order is needed. This will likely include the designation of a number of well-supported families and the removal of the subfamilial terminology. Any formal reorganization of the order, however, would be premature as long as a more complete multigene analysis remains to be performed. At lower taxonomical levels, we nevertheless propose resurrecting the genus *Spasskyellina* for three species of *Monticellia* (see above) but, for now, we consider that further nomenclatural adaptations should be delayed until clearly supported groups, reinforced by well-defined morphological characters, can be named and adequately characterized.

Results reported herein make it obvious that a new classification should not be based on the characters traditionally used for circumscribing genera and families (Rego 1994). Instead, new synapomorphies should be found to distinguish morphologically similar, but genetically distinct lineages, and to propose a more natural classification that would better reflect the evolutionary history of proteocephalideans. If applied, this would represent a clear change of strategy in our attempts to understand the evolution of the group. In practice, this could lead to the erection of numerous small genera consisting of a few species each and sharing only a few morphological, possibly discrete, synapomorphies but with good molecular support. A careful move in that direction might be the future of the systematics and taxonomy of proteocephalideans.

## Supplementary materials

Supplementary material 1 Figure 1 Data type: Phylogenetic tree Explanation note: Bayesian inference of partial (domains 1–3) 28S rDNA sequences of the complete taxon set of proteocephalideans performed using MrBayes version 3.1 using the GTR + I + G model of sequence evolution. Two parallel runs were performed for 10,000,000 generations; 8,000,000 generations were discarded as burnin. Branches with posterior probability (pp) support below 95% are collapsed; pp are indicated below branches. This dataset is made available under the Open Database License (http://opendatacommons.org/licenses/odbl/1.0/). The Open Database License (ODbL) is a license agreement intended to allow users to freely share, modify, and use this Dataset while maintaining this same freedom for others, provided that the original source and author(s) are credited.Alain de Chambrier, Andrea Waeschenbach, Makda Fisseha, Tomáš Scholz, Jean Mariaux

Supplementary material 2 Table 1 Data type: Leaf stability test results Explanation note: Leaf stability test results from the post-burnin posterior tree distribution from two MrBayes runs that included the full complement of taxa. Taxa are ranked based on their positional stability estimated from the Maximum, which is an average of all the highest percentages from all possible quartet sets for a particular taxon, Difference, which is the difference between the highest and the second highest percentages from all possible quartet sets for a particular taxon, and Entropy, which is calculated as the normalized sum of logs for each quartet percentages (except the unresolved polygamy). This dataset is made available under the Open Database License (http://opendatacommons.org/licenses/odbl/1.0/). The Open Database License (ODbL) is a license agreement intended to allow users to freely share, modify, and use this Dataset while maintaining this same freedom for others, provided that the original source and author(s) are credited.Alain de Chambrier, Andrea Waeschenbach, Makda Fisseha, Tomáš Scholz, Jean Mariaux
