# Supplementary figures and images for: A large 28S rDNA-based phylogeny confirms the limitations of established morphological characters for classification of proteocephalidean tapeworms (Platyhelminthes, Cestoda)
Source: Zookeys. 2015 Apr 27;(500):25–59. doi: 10.3897/zookeys.500.9360 (PMC4432239; doi:10.3897/zookeys.500.9360)

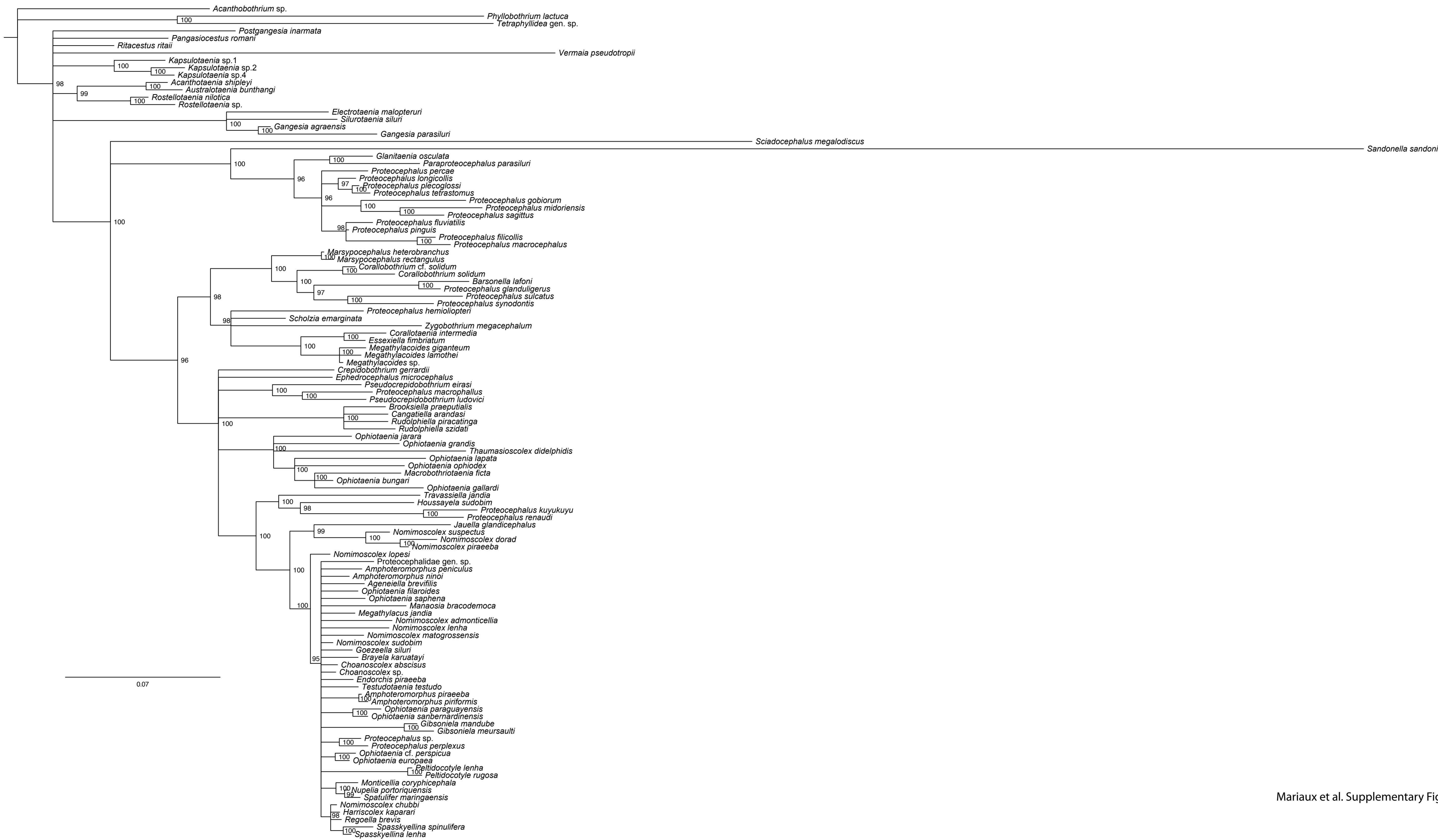

Supplement: Supplementary material 1 — Figure 1 [file zookeys-500-025-s001.pdf]
