# Supplementary material for: A large 28S rDNA-based phylogeny confirms the limitations of established morphological characters for classification of proteocephalidean tapeworms (Platyhelminthes, Cestoda)
Source: Zookeys. 2015 Apr 27;(500):25–59. doi: 10.3897/zookeys.500.9360 (PMC4432239; doi:10.3897/zookeys.500.9360)
Supplement: Supplementary material 2 — Table 1 [file zookeys-500-025-s002.docx]

**Supplementary Table 1**:

Leaf stability test results from two MrBayes runs that included the full complement of taxa

RUN 1:

-------------------------------------------------------------------------------------------------

| Taxa | Maximum | Rank | Difference | Rank | Entropy | Rank |

-------------------------------------------------------------------------------------------------

| Marsypocephalus_heterobranchus | 0.99776447 | 1 | 0.996317 | 1 | 0.99509125 | 3 |

| Marsypocephalus_rectangulus | 0.99776447 | 1 | 0.996317 | 1 | 0.99509125 | 3 |

| Barsonella_lafoni | 0.99774815 | 2 | 0.99628845 | 2 | 0.99503039 | 4 |

| Proteocephalus_glanduligerus | 0.99774815 | 2 | 0.99628845 | 2 | 0.99503039 | 4 |

| Corallobothrium_cf_solidum | 0.99774807 | 3 | 0.99628805 | 3 | 0.9950297 | 5 |

| Corallobothrium_solidum | 0.99774807 | 3 | 0.99628805 | 3 | 0.9950297 | 5 |

| Proteocephalus_sulcatus | 0.99774759 | 4 | 0.99628757 | 4 | 0.99502741 | 6 |

| Proteocephalus_synodontis | 0.99774759 | 4 | 0.99628757 | 4 | 0.99502741 | 6 |

| Corallotaenia_intermedia | 0.99751902 | 5 | 0.99591742 | 5 | 0.99470642 | 7 |

| Essexiella_fimbriatum | 0.99751902 | 5 | 0.99591742 | 5 | 0.99470642 | 7 |

| Megathylacoides_giganteum | 0.9974998 | 6 | 0.99587907 | 6 | 0.9946636 | 8 |

| Megathylacoides_lamothei | 0.9974998 | 6 | 0.99587907 | 6 | 0.9946636 | 8 |

| Megathylacoides_sp_ | 0.9974998 | 6 | 0.99587907 | 6 | 0.9946636 | 8 |

| Scholzia_emarginata | 0.99685534 | 7 | 0.9948372 | 8 | 0.99362392 | 9 |

| Zygobothrium_megacephalum | 0.99685357 | 8 | 0.99488504 | 7 | 0.99358928 | 10 |

| Proteocephalus_hemioliopteri | 0.99682044 | 9 | 0.99471299 | 9 | 0.99339507 | 11 |

| Glanitaenia_osculata | 0.99594596 | 10 | 0.99290316 | 10 | 0.99151781 | 12 |

| Paraproteocephalus_parasiluri | 0.99594596 | 10 | 0.99290316 | 10 | 0.99151781 | 12 |

| Sandonella_sandoni | 0.99576065 | 11 | 0.9925749 | 11 | 0.99090245 | 13 |

| Proteocephalus_filicollis | 0.9953593 | 12 | 0.99180775 | 12 | 0.99060635 | 14 |

| Proteocephalus_macrocephalus | 0.9953593 | 12 | 0.99180775 | 12 | 0.99060635 | 14 |

| Proteocephalus_pinguis | 0.99531605 | 13 | 0.99174046 | 13 | 0.99050985 | 15 |

| Proteocephalus_fluviatilis | 0.99531573 | 14 | 0.99173965 | 14 | 0.99050921 | 16 |

| Proteocephalus_plecoglossi | 0.99521555 | 15 | 0.99148391 | 15 | 0.99035614 | 17 |

| Proteocephalus_tetrastomus | 0.99521555 | 15 | 0.99148391 | 15 | 0.99035614 | 17 |

| Proteocephalus_gobiorum | 0.99518918 | 16 | 0.99146381 | 16 | 0.99032185 | 18 |

| Proteocephalus_midoriensis | 0.99518918 | 16 | 0.99146381 | 16 | 0.99032185 | 18 |

| Proteocephalus_sagittus | 0.99518918 | 16 | 0.99146381 | 16 | 0.99032185 | 18 |

| Proteocephalus_longicollis | 0.99518034 | 17 | 0.99142136 | 17 | 0.99022378 | 19 |

| Proteocephalus_kuyukuyu | 0.99475021 | 18 | 0.99126346 | 18 | 0.98933667 | 20 |

| Proteocephalus_renaudi | 0.99475021 | 18 | 0.99126346 | 18 | 0.98933667 | 20 |

| Houssayela_sudobim | 0.99474756 | 19 | 0.99125855 | 19 | 0.9893261 | 21 |

| Travassiella_jandia | 0.99472682 | 20 | 0.99122398 | 20 | 0.98920433 | 22 |

| Nomimoscolex_dorad | 0.99469715 | 21 | 0.99117856 | 21 | 0.98908328 | 23 |

| Nomimoscolex_piraeeba | 0.99469715 | 21 | 0.99117856 | 21 | 0.98908328 | 23 |

| Nomimoscolex_suspectus | 0.99469715 | 21 | 0.99117856 | 21 | 0.98908328 | 23 |

| Jauella_glandicephalus | 0.99460308 | 22 | 0.99102178 | 22 | 0.98864046 | 25 |

| Proteocephalus_percae | 0.99429428 | 23 | 0.98986864 | 23 | 0.98887946 | 24 |

| Nomimoscolex_lopesi | 0.99343652 | 24 | 0.98919612 | 24 | 0.98446978 | 26 |

| Acanthotaenia_shipleyi | 0.99269091 | 25 | 0.98835002 | 25 | 0.97712794 | 27 |

| Australotaenia_bunthangi | 0.99269091 | 25 | 0.98835002 | 25 | 0.97712794 | 27 |

| Rostellotaenia_nilotica | 0.99269067 | 26 | 0.98833313 | 26 | 0.97712285 | 28 |

| Rostellotaenia_sp_ | 0.99269067 | 26 | 0.98833313 | 26 | 0.97712285 | 28 |

| Kapsulotaenia_sp1 | 0.99142015 | 27 | 0.98642161 | 27 | 0.97353636 | 29 |

| Kapsulotaenia_sp2 | 0.99142015 | 27 | 0.98642161 | 27 | 0.97353636 | 29 |

| Kapsulotaenia_sp4 | 0.99142015 | 27 | 0.98642161 | 27 | 0.97353636 | 29 |

| Pangasiocestus_romani | 0.99108795 | 28 | 0.98468132 | 28 | 0.97275437 | 30 |

-------------------------------------------------------------------------------------------------

| Average | 0.98468444 | | 0.97395400 | | 0.97095876 | |

-------------------------------------------------------------------------------------------------

| Proteocephalus_macrophallus | 0.98378401 | 29 | 0.97216916 | 30 | 0.96102408 | 57 |

| Pseudocrepidobothrium_eirasi | 0.98378401 | 29 | 0.97216916 | 30 | 0.96102408 | 57 |

| Pseudocrepidobothrium_ludovici | 0.98378401 | 29 | 0.97216916 | 30 | 0.96102408 | 57 |

| Gangesia_agraensis | 0.98270805 | 30 | 0.97017796 | 31 | 0.96757754 | 31 |

| Gangesia_parasiluri | 0.98270805 | 30 | 0.97017796 | 31 | 0.96757754 | 31 |

| Electrotaenia_malopteruri | 0.98262942 | 31 | 0.97002078 | 32 | 0.96749668 | 32 |

| Silurotaenia_siluri | 0.98262942 | 31 | 0.97002078 | 32 | 0.96749668 | 32 |

| Phyllobothrium_lactuca | 0.9819081 | 32 | 0.98154124 | 29 | 0.99688733 | 2 |

| Tetraphyllidea_gen__sp_ | 0.9819081 | 32 | 0.98154124 | 29 | 0.99688733 | 2 |

| Ophiotaenia_bungari | 0.98188591 | 33 | 0.96947979 | 33 | 0.96395718 | 42 |

| Macrobothriotaenia_ficta | 0.98188583 | 34 | 0.96947963 | 34 | 0.96395676 | 43 |

| Ophiotaenia_gallardi | 0.98188575 | 35 | 0.96947947 | 35 | 0.96395626 | 44 |

| Ophiotaenia_ophiodex | 0.98183871 | 36 | 0.96939296 | 36 | 0.9638718 | 45 |

| Ophiotaenia_lapata | 0.98183855 | 37 | 0.96939264 | 37 | 0.96387089 | 46 |

| Ophiotaenia_grandis | 0.98171659 | 38 | 0.96918891 | 38 | 0.96367277 | 47 |

| Ophiotaenia_jarara | 0.98171136 | 39 | 0.96917725 | 39 | 0.96367209 | 48 |

| Proteocephalus_sp_ | 0.98146229 | 40 | 0.96749703 | 42 | 0.96545564 | 33 |

| Proteocephalus_perplexus | 0.98146229 | 40 | 0.96749703 | 42 | 0.96545564 | 33 |

| Amphoteromorphus_piraeeba | 0.98142973 | 41 | 0.96742105 | 43 | 0.96516465 | 35 |

| Amphoteromorphus_piriformis | 0.98142973 | 41 | 0.96742105 | 43 | 0.96516465 | 35 |

| Proteocephalidae_gen_sp_ | 0.98141454 | 42 | 0.96740722 | 44 | 0.96536892 | 34 |

| Amphoteromorphus_ninoi | 0.98139195 | 43 | 0.9673532 | 45 | 0.96506943 | 36 |

| Thaumasioscolex_didelphidis | 0.98137169 | 44 | 0.96858151 | 41 | 0.96322659 | 51 |

| Ageneiella_brevifilis | 0.98122166 | 45 | 0.96707832 | 46 | 0.96453717 | 39 |

| Ephedrocephalus_microcephalus | 0.98116973 | 46 | 0.96880453 | 40 | 0.95377994 | 62 |

| Testudotaenia_testudo | 0.9811641 | 47 | 0.96702043 | 47 | 0.96466032 | 38 |

| Endorchis_piraeeba | 0.98113323 | 48 | 0.96688223 | 49 | 0.96418122 | 40 |

| Ophiotaenia_cf_perspicua | 0.98112487 | 49 | 0.96698666 | 48 | 0.96492361 | 37 |

| Ophiotaenia_europaea | 0.98112487 | 49 | 0.96698666 | 48 | 0.96492361 | 37 |

| Ophiotaenia_paraguayensis | 0.98057551 | 50 | 0.96599207 | 52 | 0.96330928 | 50 |

| Ophiotaenia_sanbernardinensis | 0.98057551 | 50 | 0.96599207 | 52 | 0.96330928 | 50 |

| Amphoteromorphus_peniculus | 0.98057262 | 51 | 0.96608509 | 50 | 0.96314007 | 52 |

| Ophiotaenia_filaroides | 0.98056892 | 52 | 0.9660461 | 51 | 0.96417493 | 41 |

| Gibsoniela_mandube | 0.98056651 | 53 | 0.96584205 | 53 | 0.96339312 | 49 |

| Gibsoniela_meursaulti | 0.98056651 | 53 | 0.96584205 | 53 | 0.96339312 | 49 |

| Ophiotaenia_saphena | 0.98010253 | 54 | 0.96520811 | 54 | 0.96234631 | 53 |

| Nupelia_portoriquensis | 0.97893862 | 55 | 0.9611113 | 60 | 0.95776417 | 59 |

| Spatulifer_maringaensis | 0.97893862 | 55 | 0.9611113 | 60 | 0.95776417 | 59 |

| Monticellia_coryphicephala | 0.97893774 | 56 | 0.96110978 | 61 | 0.95775914 | 60 |

| Spasskyellina_lenha | 0.97847674 | 57 | 0.96199125 | 56 | 0.96132879 | 54 |

| Spasskyellina_spinulifera | 0.97847674 | 57 | 0.96199125 | 56 | 0.96132879 | 54 |

| Harriscolex_kaparari | 0.97840398 | 58 | 0.96188336 | 57 | 0.96120095 | 55 |

| Nomimoscolex_chubbi | 0.97840366 | 59 | 0.96188304 | 58 | 0.96120051 | 56 |

| Regoella_brevis | 0.97838541 | 60 | 0.96181711 | 59 | 0.96092986 | 58 |

| Peltidocotyle_lenha | 0.97606096 | 61 | 0.95573746 | 63 | 0.95376091 | 63 |

| Peltidocotyle_rugosa | 0.97606096 | 61 | 0.95573746 | 63 | 0.95376091 | 63 |

| Megathylacus_jandia | 0.97584219 | 62 | 0.95747919 | 62 | 0.95427925 | 61 |

| Nomimoscolex_sudobim | 0.97323073 | 63 | 0.95288207 | 65 | 0.95299441 | 64 |

| Crepidobothrium_gerrardii | 0.97294049 | 64 | 0.95045616 | 69 | 0.94358823 | 73 |

| Choanoscolex_sp | 0.97293929 | 65 | 0.95260976 | 66 | 0.95200083 | 65 |

| Ritacestus_ritaii | 0.97288622 | 66 | 0.95140284 | 67 | 0.94778561 | 70 |

| Nomimoscolex_admonticellia | 0.97224883 | 67 | 0.95337844 | 64 | 0.94804337 | 69 |

| Nomimoscolex_matogrossensis | 0.97141616 | 68 | 0.95018458 | 70 | 0.9488922 | 66 |

| Brayela_karuatayi | 0.97083834 | 69 | 0.95107996 | 68 | 0.94887415 | 67 |

| Goezeella_siluri | 0.97022861 | 70 | 0.94461103 | 78 | 0.94699861 | 71 |

| Choanoscolex_abscisus | 0.96906051 | 71 | 0.94664492 | 75 | 0.94830479 | 68 |

| Nomimoscolex_lenha | 0.96836781 | 72 | 0.94561294 | 77 | 0.94386154 | 72 |

| Postgangesia_inarmata | 0.96744525 | 73 | 0.94595262 | 76 | 0.93918871 | 74 |

| Brooksiella_praeputialis | 0.96719409 | 74 | 0.94680073 | 71 | 0.93646766 | 75 |

| Cangatiella_arandasi | 0.96719136 | 75 | 0.94679494 | 72 | 0.93646652 | 76 |

| Rudolphiella_szidati | 0.96715502 | 76 | 0.94672773 | 73 | 0.93642026 | 77 |

| Rudolphiella_piracatinga | 0.96714963 | 77 | 0.94671736 | 74 | 0.93641002 | 78 |

| Acanthobothrium_sp | 0.96452868 | 78 | 0.96452868 | 55 | 0.9986885 | 1 |

| Sciadocephalus_megalodiscus | 0.96142807 | 79 | 0.92911202 | 80 | 0.90990665 | 81 |

| Manaosia_bracodemoca | 0.96103195 | 80 | 0.93191709 | 79 | 0.93477666 | 79 |

| Vermaia_pseudotropii | 0.9543244 | 81 | 0.91954304 | 81 | 0.91791398 | 80 |

-------------------------------------------------------------------------------------------------

RUN 2:

-------------------------------------------------------------------------------------------------

| Taxa | Maximum | Rank | Difference | Rank | Entropy | Rank |

-------------------------------------------------------------------------------------------------

| Marsypocephalus_heterobranchus | 0.99751291 | 1 | 0.99578549 | 1 | 0.99470104 | 3 |

| Marsypocephalus_rectangulus | 0.99751291 | 1 | 0.99578549 | 1 | 0.99470104 | 3 |

| Barsonella_lafoni | 0.99749072 | 2 | 0.99574674 | 2 | 0.99462484 | 4 |

| Proteocephalus_glanduligerus | 0.99749072 | 2 | 0.99574674 | 2 | 0.99462484 | 4 |

| Proteocephalus_sulcatus | 0.9974904 | 3 | 0.99574625 | 3 | 0.994623 | 5 |

| Proteocephalus_synodontis | 0.9974904 | 3 | 0.99574625 | 3 | 0.994623 | 5 |

| Corallobothrium_cf_solidum | 0.99748975 | 4 | 0.99574465 | 4 | 0.99461998 | 6 |

| Corallobothrium_solidum | 0.99748975 | 4 | 0.99574465 | 4 | 0.99461998 | 6 |

| Corallotaenia_intermedia | 0.99726271 | 5 | 0.99537426 | 5 | 0.99431234 | 7 |

| Essexiella_fimbriatum | 0.99726271 | 5 | 0.99537426 | 5 | 0.99431234 | 7 |

| Megathylacoides_giganteum | 0.99724518 | 6 | 0.99533928 | 6 | 0.99427201 | 8 |

| Megathylacoides_lamothei | 0.99724518 | 6 | 0.99533928 | 6 | 0.99427201 | 8 |

| Megathylacoides_sp_ | 0.99724518 | 6 | 0.99533928 | 6 | 0.99427201 | 8 |

| Zygobothrium_megacephalum | 0.99660257 | 7 | 0.99433922 | 7 | 0.99318403 | 10 |

| Scholzia_emarginata | 0.99659083 | 8 | 0.99427088 | 8 | 0.99324572 | 9 |

| Proteocephalus_hemioliopteri | 0.99652925 | 9 | 0.99410004 | 9 | 0.99298571 | 11 |

| Glanitaenia_osculata | 0.9954955 | 10 | 0.99200826 | 10 | 0.99122433 | 12 |

| Paraproteocephalus_parasiluri | 0.9954955 | 10 | 0.99200826 | 10 | 0.99122433 | 12 |

| Sandonella_sandoni | 0.99532401 | 11 | 0.99168852 | 11 | 0.99063051 | 13 |

| Proteocephalus_filicollis | 0.99489155 | 12 | 0.99089138 | 15 | 0.99020305 | 14 |

| Proteocephalus_macrocephalus | 0.99489155 | 12 | 0.99089138 | 15 | 0.99020305 | 14 |

| Proteocephalus_fluviatilis | 0.99484814 | 13 | 0.99082473 | 16 | 0.99010641 | 16 |

| Proteocephalus_pinguis | 0.99484814 | 13 | 0.99082296 | 17 | 0.99010786 | 15 |

| Proteocephalus_plecoglossi | 0.99475624 | 14 | 0.99057397 | 20 | 0.98997346 | 17 |

| Proteocephalus_tetrastomus | 0.99475624 | 14 | 0.99057397 | 20 | 0.98997346 | 17 |

| Proteocephalus_gobiorum | 0.99474056 | 15 | 0.99057711 | 19 | 0.98995756 | 18 |

| Proteocephalus_midoriensis | 0.99474056 | 15 | 0.99057711 | 19 | 0.98995756 | 18 |

| Proteocephalus_sagittus | 0.99474056 | 15 | 0.99057711 | 19 | 0.98995756 | 18 |

| Proteocephalus_longicollis | 0.99472601 | 16 | 0.99051729 | 21 | 0.9898597 | 19 |

| Proteocephalus_kuyukuyu | 0.99457334 | 17 | 0.99092547 | 12 | 0.98920531 | 20 |

| Proteocephalus_renaudi | 0.99457334 | 17 | 0.99092547 | 12 | 0.98920531 | 20 |

| Houssayela_sudobim | 0.99457068 | 18 | 0.99092056 | 13 | 0.98919475 | 21 |

| Travassiella_jandia | 0.99456377 | 19 | 0.99090673 | 14 | 0.98915185 | 22 |

| Nomimoscolex_dorad | 0.99446062 | 20 | 0.99074055 | 18 | 0.98866549 | 23 |

| Nomimoscolex_piraeeba | 0.99446062 | 20 | 0.99074055 | 18 | 0.98866549 | 23 |

| Nomimoscolex_suspectus | 0.99446062 | 20 | 0.99074055 | 18 | 0.98866549 | 23 |

| Jauella_glandicephalus | 0.99431639 | 21 | 0.99050853 | 22 | 0.98802777 | 25 |

| Proteocephalus_percae | 0.99389519 | 22 | 0.98910471 | 25 | 0.98850748 | 24 |

| Acanthotaenia_shipleyi | 0.99370199 | 23 | 0.98990008 | 23 | 0.97995816 | 27 |

| Australotaenia_bunthangi | 0.99370199 | 23 | 0.98990008 | 23 | 0.97995816 | 27 |

| Rostellotaenia_nilotica | 0.99367739 | 24 | 0.98985112 | 24 | 0.97990273 | 28 |

| Rostellotaenia_sp_ | 0.99367739 | 24 | 0.98985112 | 24 | 0.97990273 | 28 |

| Kapsulotaenia_sp1 | 0.99249362 | 25 | 0.98791129 | 26 | 0.97654903 | 29 |

| Kapsulotaenia_sp2 | 0.99249362 | 25 | 0.98791129 | 26 | 0.97654903 | 29 |

| Kapsulotaenia_sp4 | 0.99249362 | 25 | 0.98791129 | 26 | 0.97654903 | 29 |

| Nomimoscolex_lopesi | 0.99243734 | 26 | 0.98735422 | 27 | 0.98131986 | 26 |

| Pangasiocestus_romani | 0.99226955 | 27 | 0.98643865 | 28 | 0.97624153 | 30 |

-------------------------------------------------------------------------------------------------

| Average | 0.98428871 | | 0.97319661 | | 0.97048180 | |

-------------------------------------------------------------------------------------------------

| Gangesia_agraensis | 0.98399248 | 28 | 0.97249308 | 30 | 0.96911229 | 31 |

| Gangesia_parasiluri | 0.98399248 | 28 | 0.97249308 | 30 | 0.96911229 | 31 |

| Electrotaenia_malopteruri | 0.98391465 | 29 | 0.97233751 | 31 | 0.96903146 | 32 |

| Silurotaenia_siluri | 0.98391465 | 29 | 0.97233751 | 31 | 0.96903146 | 32 |

| Proteocephalus_macrophallus | 0.983207 | 30 | 0.97085442 | 32 | 0.96040498 | 51 |

| Pseudocrepidobothrium_eirasi | 0.983207 | 30 | 0.97085442 | 32 | 0.96040498 | 51 |

| Pseudocrepidobothrium_ludovici | 0.983207 | 30 | 0.97085442 | 32 | 0.96040498 | 51 |

| Phyllobothrium_lactuca | 0.98194966 | 31 | 0.98162542 | 29 | 0.9973489 | 2 |

| Tetraphyllidea_gen__sp_ | 0.98194966 | 31 | 0.98162542 | 29 | 0.9973489 | 2 |

| Macrobothriotaenia_ficta | 0.98100781 | 32 | 0.96802162 | 33 | 0.96306314 | 40 |

| Ophiotaenia_bungari | 0.98100781 | 32 | 0.96802162 | 33 | 0.96306314 | 40 |

| Ophiotaenia_gallardi | 0.98100781 | 32 | 0.96802162 | 33 | 0.96306314 | 40 |

| Ophiotaenia_lapata | 0.98095024 | 33 | 0.96791349 | 34 | 0.96297024 | 41 |

| Ophiotaenia_ophiodex | 0.98095024 | 33 | 0.96791349 | 34 | 0.96297024 | 41 |

| Ophiotaenia_grandis | 0.98083688 | 34 | 0.96773187 | 35 | 0.96276102 | 45 |

| Ophiotaenia_jarara | 0.98083326 | 35 | 0.967719 | 36 | 0.96276417 | 44 |

| Amphoteromorphus_piraeeba | 0.98073582 | 36 | 0.96618864 | 40 | 0.96394271 | 35 |

| Amphoteromorphus_piriformis | 0.98073582 | 36 | 0.96618864 | 40 | 0.96394271 | 35 |

| Proteocephalus_sp_ | 0.98073068 | 37 | 0.96622474 | 39 | 0.96424502 | 33 |

| Proteocephalus_perplexus | 0.98073068 | 37 | 0.96622474 | 39 | 0.96424502 | 33 |

| Amphoteromorphus_ninoi | 0.98069804 | 38 | 0.96612167 | 42 | 0.96385019 | 36 |

| Proteocephalidae_gen_sp_ | 0.98067778 | 39 | 0.96612336 | 41 | 0.96415669 | 34 |

| Ageneiella_brevifilis | 0.98064023 | 40 | 0.96602551 | 43 | 0.96367447 | 38 |

| Thaumasioscolex_didelphidis | 0.98050002 | 41 | 0.96714135 | 38 | 0.96233347 | 46 |

| Endorchis_piraeeba | 0.98043988 | 42 | 0.96561685 | 46 | 0.96292329 | 42 |

| Ophiotaenia_cf_perspicua | 0.98041576 | 43 | 0.9657483 | 44 | 0.96377246 | 37 |

| Ophiotaenia_europaea | 0.98041576 | 43 | 0.9657483 | 44 | 0.96377246 | 37 |

| Testudotaenia_testudo | 0.98039325 | 44 | 0.96566172 | 45 | 0.96339407 | 39 |

| Ephedrocephalus_microcephalus | 0.9803032 | 45 | 0.96731589 | 37 | 0.95229632 | 59 |

| Amphoteromorphus_peniculus | 0.97980562 | 46 | 0.96472501 | 47 | 0.96183076 | 48 |

| Ophiotaenia_filaroides | 0.97980321 | 47 | 0.96471254 | 48 | 0.96286215 | 43 |

| Ophiotaenia_paraguayensis | 0.97972893 | 48 | 0.96445278 | 50 | 0.96166432 | 49 |

| Ophiotaenia_sanbernardinensis | 0.97972893 | 48 | 0.96445278 | 50 | 0.96166432 | 49 |

| Gibsoniela_mandube | 0.97971421 | 49 | 0.96432045 | 51 | 0.96185694 | 47 |

| Gibsoniela_meursaulti | 0.97971421 | 49 | 0.96432045 | 51 | 0.96185694 | 47 |

| Ophiotaenia_saphena | 0.97931094 | 50 | 0.96385269 | 52 | 0.96085233 | 50 |

| Nupelia_portoriquensis | 0.97858487 | 51 | 0.96057377 | 53 | 0.95694695 | 56 |

| Spatulifer_maringaensis | 0.97858487 | 51 | 0.96057377 | 53 | 0.95694695 | 56 |

| Monticellia_coryphicephala | 0.97858302 | 52 | 0.96057079 | 54 | 0.95693708 | 57 |

| Spasskyellina_lenha | 0.97763795 | 53 | 0.96033684 | 55 | 0.95984834 | 52 |

| Spasskyellina_spinulifera | 0.97763795 | 53 | 0.96033684 | 55 | 0.95984834 | 52 |

| Nomimoscolex_chubbi | 0.97756913 | 54 | 0.96023208 | 56 | 0.95972557 | 53 |

| Harriscolex_kaparari | 0.97756897 | 55 | 0.96023208 | 56 | 0.95972535 | 54 |

| Regoella_brevis | 0.97750505 | 56 | 0.96007627 | 57 | 0.95932065 | 55 |

| Peltidocotyle_lenha | 0.97515021 | 57 | 0.95404421 | 59 | 0.95273175 | 58 |

| Peltidocotyle_rugosa | 0.97515021 | 57 | 0.95404421 | 59 | 0.95273175 | 58 |

| Megathylacus_jandia | 0.97476366 | 58 | 0.9557607 | 58 | 0.95219204 | 60 |

| Ritacestus_ritaii | 0.97381819 | 59 | 0.95349743 | 60 | 0.94959397 | 64 |

| Goezeella_siluri | 0.97316601 | 60 | 0.95030268 | 64 | 0.94988725 | 63 |

| Nomimoscolex_sudobim | 0.97229635 | 61 | 0.95121189 | 63 | 0.95139385 | 61 |

| Choanoscolex_sp | 0.97227593 | 62 | 0.95121415 | 62 | 0.95129569 | 62 |

| Crepidobothrium_gerrardii | 0.97211505 | 63 | 0.9487896 | 67 | 0.94353868 | 69 |

| Nomimoscolex_admonticellia | 0.97173767 | 64 | 0.95239663 | 61 | 0.94686403 | 68 |

| Postgangesia_inarmata | 0.9701175 | 65 | 0.95010337 | 65 | 0.94204656 | 71 |

| Brayela_karuatayi | 0.97003807 | 66 | 0.94956905 | 66 | 0.94838234 | 65 |

| Nomimoscolex_matogrossensis | 0.96973923 | 67 | 0.94694826 | 68 | 0.94747094 | 66 |

| Choanoscolex_abscisus | 0.96843663 | 68 | 0.94539233 | 73 | 0.94696426 | 67 |

| Nomimoscolex_lenha | 0.96719425 | 69 | 0.94349929 | 74 | 0.94252899 | 70 |

| Brooksiella_praeputialis | 0.96637572 | 70 | 0.94589409 | 69 | 0.93566951 | 72 |

| Cangatiella_arandasi | 0.96637203 | 71 | 0.9458871 | 70 | 0.93566654 | 73 |

| Rudolphiella_szidati | 0.96633746 | 72 | 0.94582165 | 71 | 0.93562184 | 74 |

| Rudolphiella_piracatinga | 0.9663336 | 73 | 0.94581369 | 72 | 0.93561507 | 75 |

| Acanthobothrium_sp | 0.9645019 | 74 | 0.9645019 | 49 | 0.99904681 | 1 |

| Manaosia_bracodemoca | 0.96056597 | 75 | 0.93120364 | 75 | 0.93426368 | 76 |

| Vermaia_pseudotropii | 0.9577962 | 76 | 0.926025 | 76 | 0.92266058 | 77 |

| Sciadocephalus_megalodiscus | 0.94849237 | 77 | 0.90378283 | 77 | 0.88939514 | 78 |

-------------------------------------------------------------------------------------------------

Results sorted by Maximum column

* Note that the sorting in the Difference column differs from the Maximum column

** Note that the sorting in the Entropy column differs from the Maximum column
